# Drivers of Inter-individual Variation in Dengue Viral Load Dynamics

**DOI:** 10.1371/journal.pcbi.1005194

**Published:** 2016-11-17

**Authors:** Rotem Ben-Shachar, Scott Schmidler, Katia Koelle

**Affiliations:** 1 Program in Computational Biology and Bioinformatics, Duke University, Durham, North Carolina, United States of America; 2 Department of Statistical Science, Duke University, Durham, North Carolina, United States of America; 3 Department of Computer Science, Duke University, Durham, North Carolina, United States of America; 4 Department of Biology, Duke University, Durham, North Carolina, United States of America; Imperial College London, UNITED KINGDOM

## Abstract

Dengue is a vector-borne viral disease of humans that endemically circulates in many tropical and subtropical regions worldwide. Infection with dengue can result in a range of disease outcomes. A considerable amount of research has sought to improve our understanding of this variation in disease outcomes and to identify predictors of severe disease. Contributing to this research, patterns of viral load in dengue infected patients have been quantified, with analyses indicating that peak viral load levels, rates of viral load decline, and time to peak viremia are useful predictors of severe disease. Here, we take a complementary approach to understanding patterns of clinical manifestation and inter-individual variation in viral load dynamics. Specifically, we statistically fit mathematical within-host models of dengue to individual-level viral load data to test virological and immunological hypotheses explaining inter-individual variation in dengue viral load. We choose between alternative models using model selection criteria to determine which hypotheses are best supported by the data. We first show that the cellular immune response plays an important role in regulating viral load in secondary dengue infections. We then provide statistical support for the process of antibody-dependent enhancement (but not original antigenic sin) in the development of severe disease in secondary dengue infections. Finally, we show statistical support for serotype-specific differences in viral infectivity rates, with infectivity rates of dengue serotypes 2 and 3 exceeding those of serotype 1. These results contribute to our understanding of dengue viral load patterns and their relationship to the development of severe dengue disease. They further have implications for understanding how dengue transmissibility may depend on the immune status of infected individuals and the identity of the infecting serotype.

## Author Summary

Dengue is an important vector-borne disease that infects four-hundred million individuals annually. Infection results in a wide range of clinical symptoms. Though many risk factors of dengue are known, the mechanisms explaining why an individual will suffer severe symptoms are poorly understood. Clinical studies have shown characteristics of viral load kinetics of dengue-infected individuals may be indicators of disease severity. However, viral load measurements vary considerably by individual. Here we use statistical methods to empirically test hypotheses that may explain variation in dengue viral load patterns by clinical manifestation and by serotype. We show that there is statistical support for antibodies being responsible for higher disease severity during secondary dengue infections and for high viral infectivity rates of dengue serotypes 2 and 3 relative to dengue 1. These results further understanding of the relationship between viral load patterns and severe dengue disease and have important implications for dengue transmissibility.

## Introduction

Dengue is an important arthropod-borne virus whose incidence and spatial extent have increased dramatically in recent years [1]. The virus comprises 4 serotypes (DENV-1-4), each of which is further structured into clades of genetically similar viruses called genotypes [2]. Infection by any one of the four serotypes can result in a range of severity from asymptomatic infection to symptomatic dengue fever (DF) to potentially fatal dengue hemorrhagic fever (DHF). Current understanding of why some dengue-infected individuals develop severe disease while others do not manifest the infection clinically is still limited. This is due to the complex relationship between the dengue virus and the immune response [3, 4], the presence of antigenic cross-reactivity between serotypes [3, 5], phenotypic variation between dengue genotypes [6, 7], and the lack of a suitable animal model [8, 9].

Despite limitations in our current understanding of dengue pathogenesis, key risk factors for the development of severe dengue disease have been identified in longitudinal epidemiological studies [10–13]. These studies have shown that the most important risk factor for severe disease manifestation is a secondary heterologous dengue infection [10]. Clinical and experimental studies have indicated that increased disease severity during secondary infection may be explained by poorly-neutralizing antibody responses and suboptimal cross-reactive T-cell responses against the infecting serotype [3, 14–16].

Longitudinal epidemiological studies have further indicated that the identity of the infecting serotype can impact the risk of developing severe dengue disease. For example, a study in Nicaragua indicated that primary DENV-1 infections were associated with more severe clinical manifestations than primary DENV-2 infections [17]. A study in Thailand showed that DENV-2 was associated with more severe disease than DENV-1, after controlling for exposure history [11]. In this same study, secondary DENV-2 and DENV-3 infections were also twice as likely to result in DHF than secondary DENV-4 infections [11].

There is further evidence indicating that dengue genotypes can differ in virulence [18]. The Asian genotype of DENV-2 appears to cause severe disease more frequently than the American genotype of DENV-2 [19]. Very little is currently known, however, about why certain dengue serotypes and genotypes may be associated with increased disease severity. The most well-studied mechanism invoked to explain observed differences in virulence involves differences in viral replication rates between dengue viruses [10]. *In vitro* studies have shown that the Asian I genotype of DENV-2 appears to have a higher replication rate than the American genotype of this serotype [20]. Other studies have shown that dengue serotypes can differ in their ability to subvert type I interferon signaling [21] and in patterns of CD8+ T cell immunodominance [22], both of which may impact patterns of clinical manifestation.

To better understand the mechanisms underlying these important risk factors for the development of severe disease, viral load patterns of dengue-infected patients have become increasingly characterized. High viral loads are generally associated with severe dengue disease [23–25], and have been suggested to be necessary for the development of plasma leakage, a hallmark of DHF [15, 25]. Yet the relationship between high viral load and increased disease severity becomes inherently more complex when further stratified by clinical manifestation (DF or DHF) and by serotype [23]. In Vietnam, for example, where DENV-1 incidence is typically high relative to other serotypes, DENV-1-infected individuals generally appear to have higher viral loads than DENV-2-infected individuals, but DENV-2 infections are typically associated with higher disease severity [23, 26]. Apart from viral load levels, the rate of viral clearance and the time to peak viremia have been used as predictors for the development of severe dengue disease [23, 25–27].

Here, our aim is to improve our understanding of the within-host mechanisms leading to observed variation in viral load dynamics across dengue-infected patients. Insight into these mechanisms can shed light on why certain viral load features may be useful for predicting dengue disease outcome and on the mechanistic basis behind identified risk factors. Our approach is statistical in nature: we fit mechanistic within-host dengue models to viral load measurements from infected patients and use a Bayesian approach to choose between the models to identify significant drivers of viral load variation between patients. Bayesian methods have become an increasingly popular approach for parameter inference [28], in part because they allow for incorporation of prior information of parameters and for a straightforward analysis of correlation structure between parameters.

Bayesian inference via Markov chain Monte Carlo (MCMC) has been used for inference of within-host HIV models [29, 30], as well as, more recently, dengue models [31, 32]. In [31], parameters of a simple dengue within-host model were fit to viral load measurements from exclusively DENV-1 infected patients of the same clinical cohort we study. The authors considered model variants in which some parameters differed by individual while others differed only by group (defined by immune status and clinical manifestation). They found that individual variation in intrinsic incubation period and immune response parameters were needed to account for observed inter-individual variation in DENV-1 virus dynamics [31]. They further found that the parameters controlling viral infectivity and viral clearance were higher in secondary infections than in primary dengue infections [31]. In an extension of this model fit to both DENV-1 and DENV-2 viral load data along with antibody measurements [32], the authors showed that antibodies are important in controlling viral replication by two possible mechanisms: (1) antibody killing of infected cells via antibody dependent cell-mediated cytotoxicity (ADCC) or (2) antibody neutralization and clearance of virus. As in [31], some parameters were fit individually while others were allowed to vary by serotype.

To complement this work, we here consider the viral load dynamics across dengue serotypes DENV-1, DENV-2, and DENV-3, while still taking into account that variation in viral load might arise from differences in the immune status, clinical manifestation, and/or infecting serotype of patients. Specifically, we consider models of increasing complexity that reflect hypotheses that have been put forward in the dengue literature. We first consider a model in which all observed individual viral load measurements follow the same within-host dynamics. We build on this model by allowing within-host dynamics of secondary heterologous infections to differ from those of primary infections in their ability to rapidly invoke a cellular immune response. Following these models, we consider processes that have been invoked to explain differences in clinical manifestation outcomes: original antigenic sin (OAS) of T-cells and antibody dependent enhancement (ADE). Finally, we consider models that capture serotype-specific differences in viral infectivity rate, the strength of the elicited innate immune response, and the strength of the elicited T-cell response. Comparison of model fits using both the Bayesian Information Criterion (BIC) and the Deviance Information Criterion (DIC) first indicate that variation in viral load is in part explained by individual immune status (primary or secondary infection), with ADE contributing to whether a patient develops DHF in a secondary dengue infection. Variation in viral load is further explained by the identity of the infecting serotype, with the best supported hypothesis having serotypes differ in viral infectivity rate. Specifically, our analysis shows that viral infectivity rates of DENV-2 and DENV-3 are significantly higher than those of DENV-1. In the Discussion, we consider the implications of these results for understanding patterns of dengue disease severity and transmissibility.

## Methods

### Data

**Fig 1:**
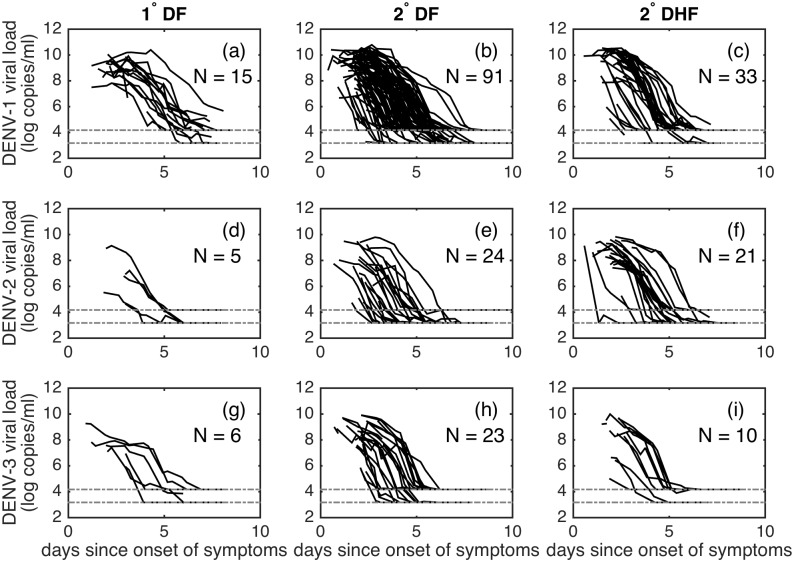
Viral load data. For all subplots, x-axes show time in days since the onset of symptoms and y-axes show viral load in log_10_ copies/ml. Dotted lines show limits of detection for the assays used. Rows correspond to serotypes: (a-c) DENV-1; (d-f) DENV-2; (g-i) DENV-3. Columns correspond to immune status and clinical manifestation: (a, d, g): primary infection dengue fever; (b, e, h): secondary infection dengue fever; (c, f, i): secondary infection dengue hemorrhagic fever. The number of patients (N) for whom viral load data are available is provided in each subplot.

We statistically analyze individual-level dengue viral load data from a clinical trial of the antiviral drug chloroquine. This trial enrolled adult dengue patients at the Hospital for Tropical Diseases in Ho Chi Minh City, Vietnam [23, 33]. Previous analyses of the viral load data indicate that chloroquine had no measurable effect on viral load dynamics [33]. (A reanalysis of the effect of chloroquine on viral load dynamics in the context of the models fit here confirms this conclusion. We provide more details on this in the Results section.) We therefore do not make a distinction between chloroquine-treated patients and control patients. Viremia of 239 dengue-infected patients was measured in the blood twice a day following hospitalization, which was within 72 hours of reported symptom onset. Virus was quantified by RT-PCR and the assay used had a limit of detection of either 1,500 copies/ml or 15,000 copies/ml [31]. Time was measured in days since the onset of symptoms. In addition to viremia measurements, the dataset included each patient’s infecting serotype, immune status (primary infection or secondary infection), and clinical manifestation (DF or DHF). Patients were infected with either DENV-1 (N = 142), DENV-2 (N = 51), DENV-3 (N = 39), or DENV-4 (N = 7). 30 of the patients were experiencing a primary infection; the remaining 209 patients were experiencing a secondary infection. Overall, 170 patients presented with DF; the remaining 69 presented with DHF. Due to the low numbers of primary infection DHF patients (N = 4) and of DENV-4-infected patients, we excluded these individuals from our analyses. Fig 1 shows the raw viral load data from the remaining 228 patients, stratified by infecting dengue serotype (DENV-1, DENV-2, or DENV-3) and by immune status and clinical manifestation (primary infection DF, secondary infection DF, or secondary infection DHF).

### Within-host dynamics

We consider dengue within-host dynamics to be governed by the following set of equations:

$$\begin{array}{ccc} dX/dt & = & -\beta XV\\ dY/dt & = & \beta XV-\alpha NY-\delta_{T}YT\\ dV/dt & = & \omega Y-\kappa V\\ dN/dt & = & qY-d_{N}N\\ dT/dt & = & q_{T}YT-d_{T}T \end{array}$$ (1)

where *X* is the number of uninfected cells, *Y* is the number of infected cells, *V* is the concentration of free virus, *N* is the number of natural killer (NK) cells, and *T* is the number of T-cells. Free virus infects target cells at rate *β*, NK cells clear infected cells at rate *α*, T-cells clear infected cells at rate *δ*_*T*_, infected cells produce free virus at rate *ω*, free virus is cleared at rate *κ*, infected cells stimulate the production of NK cells at rate *q*, NK cells decay at rate *d*_*N*_, the interaction of T-cells and infected cells stimulate the production of T-cells at rate *q*_*T*_, and T-cells decay at rate *d*_*T*_. These equations are based on a previously published within-host mathematical model for dengue that is capable of reproducing characteristic features of both primary and secondary dengue infections [34].

In both primary and secondary infections, the innate immune response (represented by NK cells) plays a pivotal role in regulating viral dynamics. This assumption is based on empirical studies that have shown support for the innate immune response (over the adaptive immune response) in clearing primary dengue infections [35, 36]. The innate immune response has further been shown to remain important for regulating viral dynamics in secondary infections [35]. In secondary infections, we assume that T-cells can contribute to the clearance of dengue-infected cells, based on a study that showed that T-cells were required for protection against heterotypic dengue infection in a mouse model [37]. In primary infections, we assume (based on [36, 38]) that T-cells contribute negligibly to viral load dynamics. We implement this assumption by setting the initial number of T-cells (*T*_0_) to zero in primary infection model fits. Note that eq (1) do not model antibody dynamics explicitly. However, the magnitude of the viral infectivity rate *β* can implicitly incorporate the role of antibodies in enhancing viral infectivity in certain subjects. Eq (1) differ from the within-host dengue equations used in [31, 32], in which the authors assume clearance of infected cells or clearance of free virus by an adaptive immune response in both primary and secondary infections.

Eq (1) contain a total of 5 initial conditions (*X*_0_, *Y*_0_, *V*_0_, *N*_0_, and *T*_0_) and 9 model parameters (*β*, *α*, *δ*_*T*_, *ω*, *κ*, *q*, *d*_*N*_, *q*_*T*_, and *d*_*T*_) (Table 1). We first perform an identifiability analysis on eq (1), similar to the approach used by Clapham and colleagues [31], to determine which of these initial conditions and parameters could in principle be independently estimated. Substituting *V*′ = *βV*, *N*′ = *αN* and *T*′ = *δ*_*T*_ *T* into eq (1) yields:

$$\begin{array}{ccc} dX/dt & = & -XV^{\prime }\\ dY/dt & = & XV^{\prime }-N^{\prime }Y-T^{\prime }Y\\ dV^{\prime }/dt & = & \beta \omega Y-\kappa V^{\prime }\\ dN^{\prime }/dt & = & q\alpha Y-d_{N}N^{\prime }\\ dT^{\prime }/dt & = & q_{T}YT^{\prime }-d_{T}T^{\prime } \end{array}$$

**Table 1:** Initial conditions and model parameters.

Parameter	Units	Estimated or assigned	Value if set	Reference
X_0_	cells ml^−1^	assigned	10^7^	[35, 39]
Y_0_	cells ml^−1^	assigned	0	
V_0_	copies ml^−1^	estimated on the log_10_ scale		
N_0_	cells ml^−1^	assigned	0	
T_0_	cells ml^−1^	assigned	10^5^	[40]
*β*	(copy ml^−1^)^−1^ day^−1^	estimated		
*κ*	day^−1^	estimated		
q	day^−1^	estimated		
*ω*	copies cell^−1^ day^−1^	assigned	10^4^	[43] (scales with *β*)
*α*	day^−1^	assigned	10^−3^	arbitrary (scales with *q*)
d_*N*_	day^−1^	assigned	0.07	[44, 45]
*δ*_*T*_	day^−1^	assigned	10^−6^	[40, 41] (fix T-cell magnitude)
*q*_*T*_	(cells ml^−1)−1^ day^−1^	estimated		
*d*_*T*_	day^−1^	assigned	0.1	[46]
IP_*g*_	days	assigned	5.9	[42]
*σ*_*I*_	log days	estimated		

Note: All model results are insensitive to changes in values of assigned parameters *X*_0_, *T*_0_, *ω*, *d*_*N*_ and *d*_*T*_ (see S4–S8 Tables).

These equations show that parameters *q* and *α* and parameters *β* and *ω* cannot be independently estimated. Due to a lack of knowledge of the value of either *q* and *α*, we assigned *α* an arbitrary value of 10^−3^ per day for all analyses. We further assign *ω* a value of 10^4^ copies/cell/day based on a virological study (Table 1).

We assign values to initial conditions and parameters that have been independently derived in the literature. Trivially, we set the initial number of infected target cells (*Y*_0_) and NK cells (*N*_0_) both to 0. Following [31], we set the initial number of uninfected target cells (*X*_0_) to 10^7^ cells/ml (Table 1). This value is reasonable, given the range of healthy monocyte densities in plasma of adults [35, 39]. We further set death rates of NK and T-cells (*d*_*N*_ and *d*_*T*_, respectively) based on literature estimates (Table 1).

The identifiability analysis further shows that without T-cell data we cannot estimate *T*_0_ and *δ*_*T*_ independently. We therefore first set *T*_0_ to a value of 10^5^ cells/mL, a reasonable value based on immunological studies (Table 1) and attempted to fit *δ*_*T*_. However, due to lack of T-cell data and early viral load data, the parameter *δ*_*T*_ became practically unidentifiable (see Results section below), covarying almost perfectly with the parameter *q*_*T*_. We thus set *δ*_*T*_ to 10^−6^ per day such that T-cell counts would reach maximum values on the order of 10^6^ cells/ml, consistent with values from studies examining T-cell dynamics in dengue infections [40, 41].

The only initial condition we therefore fit in our statistical analysis of the viral load data was the initial amount of free virus *V*_0_, which we estimate on the log-transformed scale. The parameters in eq 1 we fit were *β*, *κ*, *q*, and *q*_*T*_.

Because the viral load data were reported as a function of time since the onset of symptoms and eq (1) describe within-host dengue dynamics from the start of infection, it was necessary to further estimate the incubation period (IP), defined as the time between viral inoculation and the onset of symptoms. Since IP correlates strongly with the initial amount of free virus (*V*_0_), we do not attempt to estimate IP. Instead, we rely on results from a study that used Bayesian time-to-event models to estimate IP durations using observations from 35 empirical dengue studies [42]. This study estimated an IP of 5.9 days for dengue, with 95% of the estimates lying between 3 and 10 days. Examination of the viral load data shown in Fig 1 suggests that much of the inter-individual variability of the data may be explained by individual differences in IP. We therefore include a random effect on the IP to account for this variability. To incorporate this random effect, we assume that individual incubation periods are log-normally distributed based on the results in [42]. We set the mean value of the log-normal distribution to *log*(*IP*_*g*_) where *IP*_*g*_ is 5.9 days [42] and estimate the standard deviation of the incubation period, *σ*_*I*_.

### Bayesian implementation

To fit eq (1) to the viral load data shown in Fig 1, we assume log_10_ viremia measurements have normally-distributed measurement errors.

Given an incubation period *IP*_*j*_ for individual *j*, the likelihood of the model parameters *γ* given this individual’s viral load data *D*_*j*_ is given by:

$$\begin{array}{cc} L_{j}\left(\gamma |D_{j}\right) & =\prod_{k=1}^{n}\phi \left(D_{j}\left(t_{k}\right)|M\left(\gamma ,t_{k}+IP_{j}\right),\sigma_{\epsilon }\right)1_{D_{j(t_{k})}>LOD}+\\ & \Phi \left(LOD|M\left(\gamma ,t_{k}+IP_{j}\right),\sigma_{\epsilon }\right)1_{D_{j(t_{k})}\leq LOD} \end{array}$$ (2)

The likelihood is evaluated over the number of viral load data points *n* of individual *j*. A given viral load data point *k* is provided at time *t*_*k*_, measured in days since the onset of symptoms. D_*j*_(*t*_*k*_) is thus the viremia measurement from individual *j* at *t*_*k*_ days since the onset of symptoms. To account for the incubation period, the model-predicted viremia measurements *M* are measured at times *t*_*k*_ + IP_*j*_. Both D_*j*_ and *M* viremia levels are on the log_10_ scale. *ϕ* is the Gaussian probability density function (pdf), where the parameter quantifying the standard deviation of the measurement error is *σ*_*ϵ*_, which, as in [31, 32], we set to 1. We use the Gaussian pdf to calculate the probability of observing *D*_*j*_(*t*_*k*_) only if the data point *D*_*j*_(*t*_*k*_) lies above the limit of detection (LOD). Φ is the Gaussian cumulative distribution function (cdf). We use the Gaussian cdf to calculate the probability of observing a data point *D*_*j*_(*t*_*k*_) at or below the LOD. Rather than sample the *IP*_*j*_s individually, we integrate them out numerically, assuming the probability density of *IP*_*j*_ is given by $logN(log\left(IP_{g}\right),\sigma_{I}^{2})$. The overall log-likelihood is then the sum of log-likelihoods over all individuals for whom we have viral load data. The full log-likelihood expression is provided in the S1 Text.

We use the Metropolis-Hastings algorithm for MCMC parameter estimation with a multivariate normal proposal distribution truncated to the positive quadrant as the model parameters shown in Table 1 are restricted to be positive. Because values of *V*_0_ are close to 0 but cannot be 0, instead of estimating *V*_0_ directly we estimate *log*_10_(*V*_0_). We use uniform improper priors for all estimated parameters.

The MCMC algorithm was run for a total of 300,000 iterations, with a burn-in of 150,000 iterations. Each model we considered was run for 4 different sets of initial conditions of all parameters estimated. We assessed model convergence by calculating the Rubin-Gelman convergence diagnostic for each model and ensuring that $\hat{R}<1.1$ for each parameter [47], as well as assessing posterior trace plots for convergence by eye. For all models, we found $\hat{R}$ to be < 1.1 for all parameters. To incorporate parameter correlations, after 30,000 iterations, we began adapting the covariance of the proposal to the model posterior. At 150,000 iterations, we fixed the covariance matrix.

In some of the figures shown in the Results section, we plot estimates of individual incubation periods. Since for each individual *j* we marginalize over the individual incubation periods during the process of parameter estimation (see above and the full likelihood expression provided in the supplemental material), our plotting of individual incubation periods is simply to show the most likely incubation periods for individuals of a certain group relative to individuals of other groups. Draws from the joint posterior are obtained by sampling from the conditional distributions of the incubation periods given sampled parameters *γ*, proportional to *p*(*IP*_*j*_|*logN*(*log*(*IP*_*g*_), *σ*_*I*_))*L*_*j*_(*γ*|*D*_*j*_).

### Models considered

We estimate parameters of eq (1) for models of varying complexity that reflect hypotheses that have been put forward by virological and immunological studies of dengue infection. Our goal in fitting these various models is to determine which hypotheses best explain variation in viral load patterns observed in dengue infected patients. Table 2 summarizes the set of models we consider.

**Table 2:** Models considered. Column *k* lists the number of free parameters. Global parameters are shared across all individuals, regardless of clinical manifestation or infecting serotype. Also shown are the median log-likelihood values, BIC and DIC values for all models considered.

Model	*k*	Global parameters	Clinical-manifestation-specific parameters	Serotype-specific parameters	Log-likelihood	BIC	DIC
0	5	*V*_0_, *β*, *κ*, *q*, *σ*_*I*_	–	–	-2411	4857	4828
1	6	*V*_0_, *β*, *κ*, *q*, *q*_*T*_, *σ*_*I*_	–	–	-2346	4733	4698
*OAS*_1_	7	*V*_0_, *β*, *κ*, *q*, *q*_*T*_, *σ*_*I*_	*δ*_*T*_	–	-2347	4741	4700
*OAS*_2_	8	*V*_0_, *β*, *κ*, *q*, *σ*_*I*_	*δ*_*T*_, *q*_*T*_	–	-2346	4746	4699
*ADE*	7	*V*_0_, *κ*, *q*, *q*_*T*_, *σ*_*I*_	*β*	–	-2346	4740	4699
SS_*β*_	8	*V*_0_, *κ*, *q*, *q*_*T*_, *σ*_*I*_	–	*β*	-2332	4720	4673
SS_*q*_	8	*V*_0_, *β*, *κ*, *q*_*T*_, *σ*_*I*_	–	*q*	-2345	4745	4699
${\text{SS}}_{q_{T}}$	8	*V*_0_, *β*, *κ*, *q*, *σ*_*I*_	–	*q*_*T*_	-2337	4728	4681
${\text{SS}}_{\beta_{ADE}}$	11	*V*_0_, *κ*, *q*, *q*_*T*_, *σ*_*I*_	*β*	*β*	-2334	4744	4679

The first model we fit (model 0) is the most basic dengue model: it assumes that viral load dynamics are regulated by the innate immune response, and that the adaptive immune response plays a negligible role in regulating viral dynamics in both primary and secondary dengue infections (*T*_0_ = 0, *q*_*T*_ is not estimated). It further assumes that viral load dynamics do not differ by the infecting serotype or by disease manifestation (DF/DHF). For this model we fit four parameters (*β*, *κ*, *q*, *σ*_*I*_) and one initial condition *V*_0_.

The second model we fit (model 1) assumes that T-cells are important in clearing infected cells during secondary infections, but not in primary infections. We implement this model by fitting the primary infection data to eq (1) under the assumption that *T*_0_ = 0, and by fitting the secondary infection data to eq (1) under the assumption that *T*_0_ = 10^5^ cells/ml. Under this model, we assume that all other parameters and initial conditions listed in Table 1 are the same across infection types. For this model we therefore fit five parameters (*β*, *κ*, *q*, *q*_*T*_, *σ*_*I*_) and one initial condition *V*_0_.

#### Models considering differences in clinical manifestation

The next models we consider aim to address whether there are differences in viral load dynamics between individuals presenting with different clinical manifestations (DF vs DHF), and, if so, the mechanism(s) responsible for these differences. We first consider models that incorporate the process of original antigenic sin of T-cells (model OAS_1_ and model OAS_2_). OAS proposes that fast-acting memory T-cell populations cause a strong pro-inflammatory response during secondary heterologous dengue infections. Yet, because these populations have low avidity to the infecting (secondary) virus, the protective effects of the T-cells in lysing infected cells are suboptimal [48]. In support of OAS, a study of T-cell responses in Thai children showed that the majority of dengue virus-specific T-cells had low affinity to the infecting serotype [16].

We formulate two variants of the OAS model. First, we consider a model (OAS_1_) in which we allow the rate of T-cell lysis of infected cells (*δ*_*T*_) to vary by clinical manifestation (DF or DHF) during a secondary infection, with the expectation that *δ*_*T*_ would be lower in DHF-manifesting individuals relative to DF-manifesting individuals. Second, we consider a model (OAS_2_) in which in addition to the T-cell lysis rate *δ*_*T*_, the T-cell activation rate *q*_*T*_ also varies by clinical manifestation. For model OAS_2_ we expect *q*_*T*_ will be higher in DHF-manifesting individuals relative to DF-manifesting individuals. Note that in model 1 we fixed the value of *δ*_*T*_ at 10^−6^ per day. In fitting both the *OAS*_1_ model and the *OAS*_2_ model, we therefore chose to fix $\delta_{T_{DHF}}$ at this value and estimated $\delta_{T_{DF}}$. For model *OAS*_2_ we parameterize $q_{T_{DHF}}\;=\;q_{T_{DF}}\;+\;\Delta_{q_{T}}$ and estimate $q_{T_{DF}}$ and $\Delta_{q_{T}}$, where $\Delta_{q_{T}}$ may be positive or negative.

We next consider a model that incorporates the process of antibody dependent enhancement (model ADE). ADE proposes that antibodies produced in a primary infection cannot completely neutralize virus present in a secondary heterologous infection. However, the resulting partially neutralized immune complexes can enter Fc-*γ*-bearing target cells, resulting in increased viral infectivity [4, 10]. Studies have shown that human dengue antibodies can enhance viral replication *in vitro* [14, 49] and *in vivo* [50]. The ADE model therefore hypothesizes that the viral infectivity rate *β* is higher in DHF-manifesting individuals relative to DF-manifesting individuals. The model is mathematically formulated by allowing *β* to vary by clinical manifestation (DF/DHF) during a secondary infection. We assume the same *β* value for primary DF and secondary DF individuals. We parameterize *β* values for secondary DHF cases by letting *β*_*DHF*_ = *β*_*DF*_ + Δ_*β*_, and estimating Δ_*β*_ and *β*_*DF*_, where Δ_*β*_ may be positive or negative.

#### Models considering differences between dengue serotypes

We also consider models that aim to address whether there are differences in viral load dynamics between individuals as a function of their infecting serotypes, and, if so, the mechanism(s) responsible for these differences. These models do not include variation in any parameter by clinical manifestation. Our first serotype-specific model is motivated by DENV-2 infections, which often, though not always [51], result in more severe disease than other serotypes [10]. *In vitro* studies have shown that the DENV-2 genotypes that cause more severe disease have higher replication rates than those that cause milder disease [18]. We therefore first explore serotype-specific differences in the viral replication rate by allowing the viral infectivity rate *β* to differ by serotype (model SS_*β*_). Due to problems of identifiability discussed above, we do not consider a model that varies the viral replication rate (*ω*).

Our second serotype-specific model is motivated by a recent *in vitro* study showing that dengue serotypes can differ in their ability to block type I interferon signaling [21]. Specifically, Medina and coauthors found that DENV-3 showed the lowest level of inhibiting interferon signaling, followed by DENV-1, and DENV-2. To test this hypothesis, we fit a model that allows the rate at which the innate immune response is elicited (*q*) to vary by serotype (model *SS*_*q*_).

Our third and final serotype-specific model is motivated by multiple *in vivo* and *in vitro* studies that suggest that there may be intrinsic differences in the reactivity of CD8+ T-cell responses to infecting serotypes [22, 52, 53]. In order to investigate differences in the effector functions of T-cells by infecting serotype, we fit a model (model ${SS}_{q_{T}}$) that allows for serotype-specific differences in the rate at which T-cell responses are elicited (*q*_*T*_).

As for the clinical manifestation models, we reparameterize serotype-specific parameters for DENV-2 and DENV-3 using DENV-1’s parameter value as a reference point. For example, for model *SS*_*β*_, we let $\beta_{2}\;=\;\Delta_{\beta_{2}}\;+\;\beta_{1}$ and $\beta_{3}\;=\;\Delta_{\beta_{3}}\;+\;\beta_{1}$, and we estimate *β*_1_, $\Delta_{\beta_{2}}$ and $\Delta_{\beta_{3}}$.

#### Model considering differences by clinical manifestation and between dengue serotypes

Finally, we consider a model in which parameters vary both by clinical manifestation and between dengue serotypes (model ${SS}_{\beta_{ADE}}$). We allow *β* to vary by serotype and additionally allow *β* to vary by clinical manifestation, fitting a total of 11 parameters, where 6 of these parameters are viral infectivity parameters.

As for the serotype-specific models, we reparameterize serotype-specific parameters for DENV-2 and DENV-3 using DENV-1’s parameter value as a reference point. For secondary infection DHF, we reparameterize each serotype by an additional DHF parameter. For example, we let $\beta_{2,DF}\;=\;\Delta_{\beta_{2}}\;+\;\beta_{1}$ and $\beta_{2,DHF}\;=\;\beta_{2,DF}\;+\;\Delta_{\beta }{}_{{}_{2},DHF}$ and we estimate *β*_1_, $\Delta_{\beta_{2}}$ and $\Delta_{\beta }{}_{{}_{2},DHF}$.

### Model selection

We select between models of increasing complexity by using two criteria for model selection: the Bayesian Information Criterion (BIC) [54] and the Deviance Information Criterion (DIC) [55]. BIC is a function of the highest posterior log-likelihood and explicitly penalizes more complex models with higher number of parameters more strongly than other model selection criteria, such as the Akaike Information Criterion (AIC) [54]. It is calculated as: *BIC* = −2*ln*(*L*) + *kln*(*n*), where *ln*(*L*) is the highest log-likelihood of the model samples, *k* is the number of free model parameters to be estimated, and *n* is the total number of viral load measurements (*n* = 2415 data points). A difference in BIC between two models of 2–6 is considered positive, 6–10 strong and > 10 very strong support in favor of the model with lower BIC [54]. DIC is a model criterion typically used for Bayesian hierarchical models and is frequently used for model selection in MCMC analysis. Though DIC does not penalize complex models with more parameters explicitly, it is considered a Bayesian analogue of AIC [55]. DIC is defined as the difference between the posterior mean of the deviance and the deviance of the posterior mean of the parameters. Mathematically this is described as: $D\left(\theta \right)=-2ln\left(\bar{L(\theta )}\right)+2ln\left(L\left(\bar{\theta }\right)\right)$, where bar denotes expectation with respect to the posterior. In more complex models the deviance of the posterior means of the parameters is expected to be higher. A difference in DIC of 1–2 values is considered positive, whereas a difference > 3 is considered strong support for the model with lower DIC [55].

## Results

To first determine whether the viral load data provide statistical support for the role of T-cells in clearing infected target cells in secondary dengue infections, we compare the results of fitting models 0 and 1 to the data (Table 2). The median log-likelihood for model 1 is significantly higher than that of model 0, indicating that the inclusion of T-cell dynamics in secondary infections significantly improves model fit. The BIC and DIC values of these models indicate that model 1 is preferred over model 0, despite its higher level of model complexity. Traces of model 1’s MCMC runs with different initial conditions are shown in S1 Fig, visually indicating convergence. The correlation structure of model 1 marginal posteriors are shown in S2 Fig. Median likelihood estimates and 95% posterior credible intervals for model 1 are given in Table 3. S3 Fig shows that model 1 results are insensitive to chosen values of *δ*_*T*_, the rate of T-cell lysis, with *q*_*T*_ estimates compensating for changes in *δ*_*T*_ (see also S1 Text). For model 1, we further re-evaluated whether chloroquine treatment had a measurable effect on viral load dynamics, with results indicating that it did not (S1 Text, S1 Table, S4 Fig). For sake of completeness, parameter values for model 0 are provided in S2 Table.

**Table 3:** Parameter estimates for model 1 and model SS_*β*_. Median marginal posterior parameter estimates are reported, with 95% posterior credible intervals for each parameter in parentheses.

Model	*log*_10_ *V*_0_ (cells/ml)	*β*×10^−10^ ((copy/ml)^−1^ d^−1^)	*κ* (d^−1^)	*q*×10^−4^ (d^−1^)	*q*_*T*_×10^−6^ (d^−1^)	*σ*_*I*_ (log days)
1	-3.2 (-4.8, -1.8)	4.6(4.8, 5.3)	5.2 (5.0, 5.9)	6.5(5.5, 8.0)	1(0.9, 1.2)	0.2 (0.18, 0.22)
SS_*β*_	-3.4 (-5.0, -2.0)	*β*_1_: 4.5 (3.9, 5.1) *β*_2_: 5.1 (4.4, 6.0) *β*_3_: 5.1 (4.4, 6.0)	5.2 (4.8, 5.8)	6.6 (5.6, 7.9)	1 (9.1, 1.2)	0.19 (0.17, 0.21)

**Fig 2:**
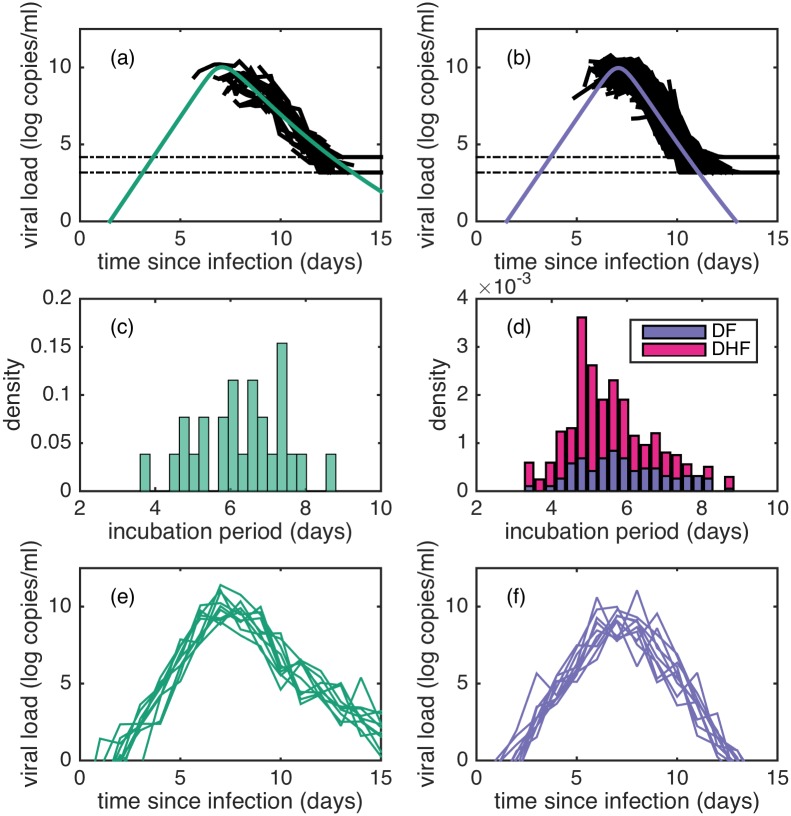
Fits of model 1 to viral load data and forward simulations of model 1. (a) Simulation of model 1 (green) using median log-likelihood estimates, plotted alongside viral load measurements from all individuals experiencing a primary infection. (b) Simulation of model 1 (purple) using median log-likelihood estimates, plotted alongside viral load measurements from all individuals experiencing a secondary infection. In (a) and (b), viremia measurements are shifted in time based on sampled estimates of individual *IP* values as described in Methods. Dotted lines indicate the limits of detection. (c) Histogram showing sampled incubation period estimates for individuals experiencing a primary infection. (d) Histogram showing sampled incubation period estimates for individuals experiencing a secondary infection, stratified by clinical manifestation (DF or DHF). (e) 10 forward simulations of model 1 in the presence of observation noise for individuals experiencing a primary infection. (f) 10 forward simulations of model 1 in the presence of observation noise for individuals experiencing a secondary infection.

Fig 2 shows the fit of model 1 to the viral load data, along with forward simulations of the model. Fig 2a and 2b show the fits to all individuals experiencing primary and secondary infections, respectively, where viral load trajectories have been shifted in time according to sampled individual incubation periods. These figures show that much of the variation in viral load dynamics apparent in Fig 1 can be accounted for by inter-individual variation in IP. Fig 2c and 2d show distributions of sampled incubation periods for individuals experiencing primary and secondary dengue infections, respectively. Intriguingly, primary infection incubation periods appear to generally be longer than secondary infection incubation periods. Fig 2e and 2f show forward simulations of model 1, including measurement noise. These simulations indicate that model-predicted viral load dynamics can quantitatively reproduce the degree of inter-individual variation present in observed viral load dynamics shown in Fig 2a and 2b.

**Fig 3:**
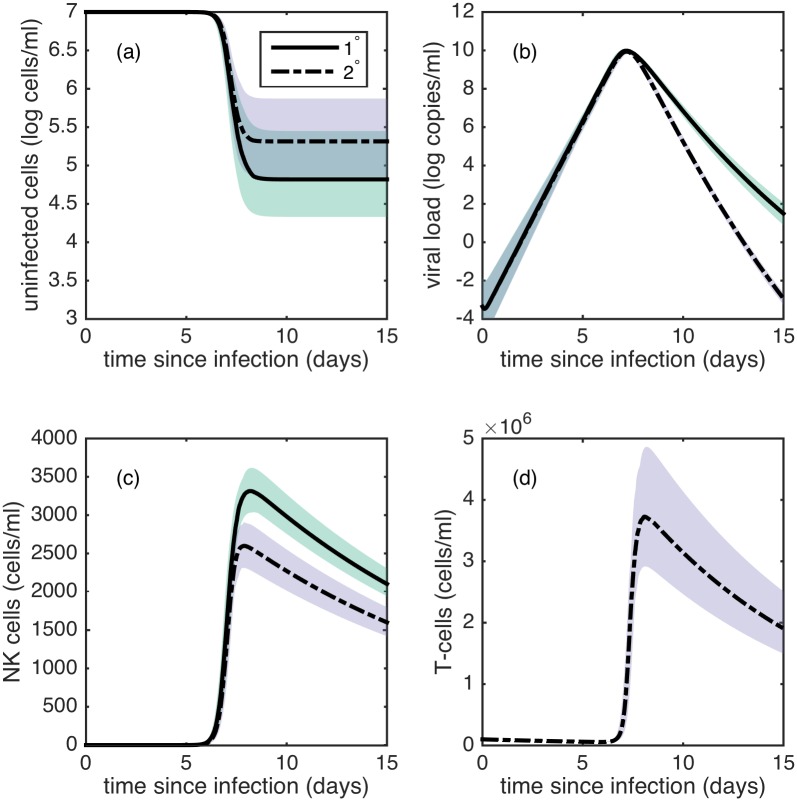
Forward simulations of model 1. (a) Dynamics of uninfected target cells *X*. (b) Dynamics of free virus *V*. (c) Dynamics of NK cells *N*. (d) Dynamics of T-cells *T* in secondary infections. In (a)-(d), black solid and dashed lines show simulations of primary and secondary infections, respectively using median likelihood parameter estimates. Green and purple shaded regions show 95% posterior credible intervals of primary and secondary infections, respectively. Dark green regions show overlap in credible interval regions. Credible intervals were constructed from 100 simulations of model 1, where parameters were sampled from the posterior for each simulation. Subplot (d) shows only secondary infection dynamics as T-cells are not modeled in primary infection dynamics.

Fig 3 shows forward simulations of model 1’s dynamics in the absence of observation noise, with 95% posterior credible intervals. The secondary infection model simulations show a steeper viral decline compared to primary infection simulations (Fig 3b) due to the activation of T-cells, consistent with empirical observations [24, 25]. Although some viral kinetic studies have shown that secondary infections have higher viral peaks than primary infections [23–25, 56], simulations of model 1 do not reproduce this finding (Fig 3b). This may be because viral peaks were present in under 30% of individuals. Our model simulations further indicate that we expect NK-cell absolute counts to be lower in secondary infection relative to primary infections (Fig 3c), consistent with findings in [35].

### Models considering differences in clinical manifestation

**Fig 4:**
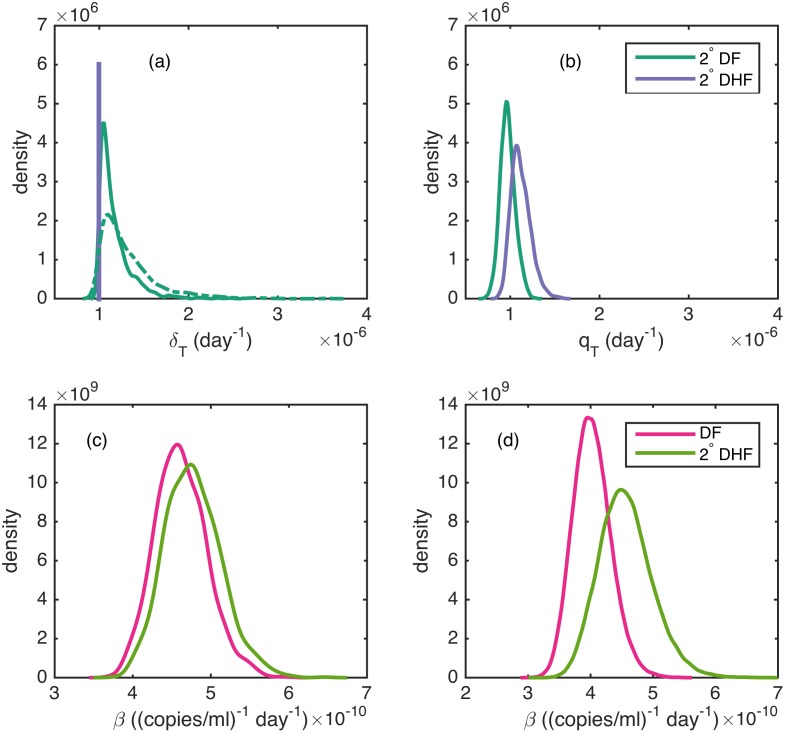
Parameter estimates from the clinical manifestation models. (a) Density estimates of T-cell clearance rate $\delta_{T_{DF}}$ for models *OAS*_1_ (solid green) and *OAS*_2_ (dashed green), alongside the assigned value of $\delta_{T_{DHF}}$ (purple line). (b) Density estimates of T-cell activation rates $q_{T_{DF}}$ and $q_{T_{DHF}}$ for model *OAS*_2_. (c) Density estimates of viral infectivity rates *β*_DF_ and $\beta_{{SI}_{DHF}}$ for model *ADE*. (d) Density estimates of *β*_*DF*_ and $\beta_{{SI}_{DHF}}$ when the ADE model is fitted to the subset of the data for individuals with a detected viral peak. The median parameter estimate and 95% posterior credible interval (in parentheses) of the difference of the parameter estimates shown in (a-d) are: (a) model *OAS*_1_: $\delta_{T_{DF}}-\delta_{T_{DHF}}$ 1.1 × 10^−7^(3.8 × 10^−9^, 6.5 × 10^−7^). model *OAS*_2_: $\delta_{T_{DF}}-\delta_{T_{DHF}}$: 2.1 × 10^−7^(7.2 × 10^−9^, 1.1 × 10^−6^). (b) $q_{T_{DHF}}-q_{T_{DF}}$: 1.4 × 10^−7^(2.8 × 10^−8^, 3.1 × 10^−7^). (c) *β*_2^*o*^, *DHF*_ − *β*_*DF*_: 1.4 × 10^−11^(9.9 × 10^−13^, 3.8 × 10^−11^). (d) *β*_2^*o*^, *DHF*_ − *β*_*DF*_: 5.2 × 10^−11^(1.7 × 10^−11^, 1.0 × 10^−10^).

To determine if original antigenic sin of T-cells can account for differences in viral dynamics between secondary DF and DHF patients, we fit models *OAS*_1_ and *OAS*_2_ (described above), with our expectation being that $\delta_{T_{DF}}>\delta_{T_{DHF}}$ for both models and $q_{T_{DHF}}>q_{T_{DF}}$ for model *OAS*_2_. Fig 4a shows the density estimates for $\delta_{T_{DF}}$, relative to the assigned value of 10^−6^ per day for $\delta_{T_{DHF}}$ for models *OAS*_1_ and *OAS*_2_. For both models, $\delta_{T_{DF}}$ estimates do not appreciably differ from the value assigned to $\delta_{{T,}_{DHF}}$. For model *OAS*_1_, the median log-likelihood is not higher than that of model 1, despite an additional free parameter (Table 2). The BIC and DIC values for model *OAS*_1_ are higher than that of model 1, indicating that model 1 is statistically preferred over model *OAS*_1_ (Table 2). For model *OAS*_2_, Fig 4b shows the density estimates for $q_{T_{DF}}$ and $q_{T_{DHF}}$. Though $q_{T_{DHF}}>q_{T_{DF}}$ as expected, the median posterior log-likelihood of model *OAS*_2_ is the same value as that of model 1, despite two additional free parameters (Table 2). Furthermore, the BIC and DIC values for model *OAS*_2_ are higher than that of model 1, indicating that model 1 is statistically preferred over model *OAS*_2_ (Table 2). For sake of completeness, S2 Table shows parameter estimates for models *OAS*_1_ and *OAS*_2_.

To determine if antibody-dependent enhancement (ADE) can account for differences in viral dynamics between individuals that differ in clinical manifestation, we fit model ADE (described above), with the expectation that the viral infectivity rate in DHF-manifesting individuals would exceed the viral infectivity rate in DF-manifesting individuals: $\beta_{SI_{DHF}}>\beta_{DF}$. Fig 4c shows the density estimates for parameters $\beta_{{SI}_{DHF}}$ and *β*_*DF*_. While $\beta_{{SI}_{DHF}}$ estimates do appear to be higher than *β*_*DF*_ estimates, the difference is small. As a result, the median log-likelihood of model ADE does not differ from that of model 1 (Table 2). Due to the higher number of free parameters in model ADE, this model therefore results in a higher BIC and DIC than model 1, such that model 1 is statistically preferred over model ADE. Again, for sake of completeness, S2 Table shows parameter estimates for model *ADE*.

One reason why we might find only slight differences in viral infectivity rates between DHF-manifesting and DF-manifesting individuals is because most of the viral load data shown in Fig 1 do not include the viral peak. In the absence of viral peaks, the model cannot disentangle higher viral infectivity rates *β* from lower incubation periods *IP* for DHF compared to DF patients. To see if we could disentangle differences in *β* from differences in *IP*, we fit model 1 and model ADE to the subset of the viral load data in which viral peaks were present (N = 65 individuals). Instead of fitting *log*(*V*_0_) and setting *IP*_*g*_ to 5.9 days, we fit *IP*_*g*_ and set *log*(*V*_0_) = −3.2 log copies/ml, the median posterior value of *log*(*V*_0_) for model 1 (Table 3). We adopted this approach because we expect that in these subjects the mean *IP* value of individuals is lower than in the full dataset. We incorporated a lognormal prior on *IP*_*g*_ such that *IP*_*g*_ ∼ *logN*(*log*(5.9), 0.15), to ensure that the mean *IP*_*g*_ value is between the expected 3–10 days [42]. Table 4 shows that the median posterior log-likelihood for model ADE in this case is higher than that of model 1, and that the BIC and DIC of model ADE are significantly lower than that of model 1. This indicates that model ADE is preferred over model 1 when only viral load data containing viral peaks is considered. Parameter values for model 1 and model ADE fits to this subset of the data are provided in S3 Table.

**Table 4:** Models fit to subset of the data containing viral peaks (N = 65 individuals, n = 747 viral load data points). The parameters estimated, the median log-likelihood values, BIC and DIC values for each model are given.

Model	*k*	Global parameters	CM-specific parameters	Serotype-specific parameters	Log-likelihood	BIC	DIC
1	6	*β*, *κ*, *q*, *q*_*T*_, *IP*_*g*_, *σ*_*I*_	–	–	-863	1760	1730
*ADE*	7	*κ*, *q*, *q*_*T*_, *IP*_*g*_, *σ*_*I*_	*β*	–	-859	1756	1724
SS_*β*_	8	*κ*, *q*, *q*_*T*_, *IP*_*g*_, *σ*_*I*_	–	*β*	-853	1749	1711
${\text{SS}}_{q_{T}}$	8	*β*, *κ*, *q*, *IP*_*g*_, *σ*_*I*_	–	*q*_*T*_	-857	1757	1718
${\text{SS}}_{\beta_{ADE}}$	11	*κ*, *q*, *IP*_*g*_, *q*_*T*_, *σ*_*I*_	*β*	*β*	-852	1766	1713

Fig 4d shows density estimates for model ADE’s $\beta_{{SI}_{DHF}}$ and *β*_*DF*_ on this subset of the data. Estimates for $\beta_{{SI}_{DHF}}$ exceed those of *β*_*DF*_, as expected under ADE. S5 Fig shows simulations of model ADE fit to this data subset. Viral loads peak earlier in DHF relative to DF patients (S5b Fig), and immune cells are activated earlier in DHF relative to DF patients (S5c and S5d Fig), consistent with the findings in [35].

### Models considering differences between dengue serotypes

**Fig 5:**
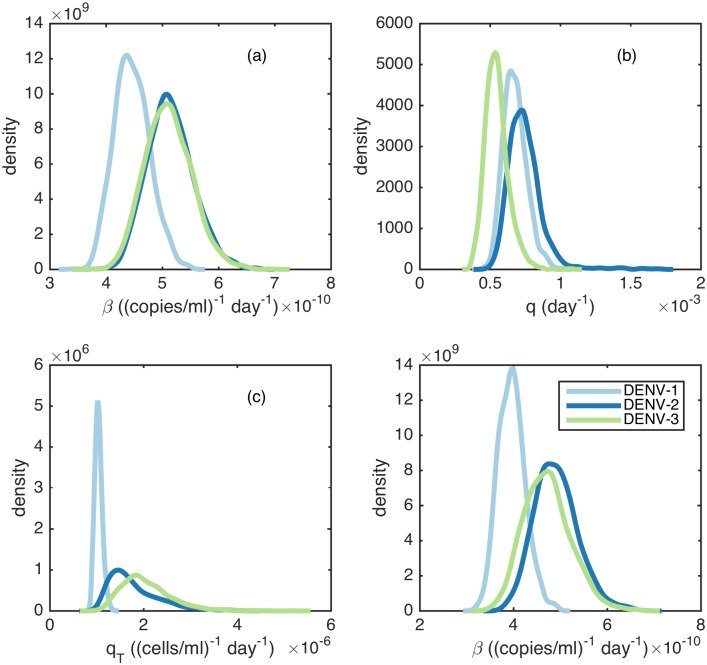
Parameter estimates from the serotype-specific models. (a) Density estimates for serotype-specific viral infectivity rates *β* for model SS_*β*_. (b) Density estimates for serotype-specific innate immune response activation rates for model SS_*q*_. (c) Density estimates for serotype-specific T-cell activation rates *q*_*T*_ for model ${\text{SS}}_{q_{T}}$. (d) Density estimates for serotype-specific viral infectivity rates *β* for model SS_*β*_ that was fitted to only the subset of the viral load data with individuals with a detected viral peak. The median parameter estimate and 95% posterior credible interval (in parentheses) of the difference of the parameter estimates shown in (a-d) relative to DENV-1 are: (a) *β*_2_ − *β*_1_: 6.6 × 10^−11^(3.6 × 10^−11^, 1.1 × 10^−10^), *β*_3_ − *β*_1_: 6.4 × 10^−11^(3.1 × 10^−11^, 1.1 × 10^−10^). (b) *q*_2_ − *q*_1_: 5.2 × 10^−5^(−1.5 × 10^−4^, 4.1 × 10^−4^), *q*_3_ − *q*_1_: −1.3 × 10^−4^(−3.1 × 10^−4^, 8.0 × 10^−5^). (c) $q_{T_{2}}-q_{T_{1}}$: 6.1 × 10^−7^(1.3 × 10^−7^, 2.2 × 10^−6^), $q_{T_{3}}-q_{T_{1}}$: 9.3 × 10^−7^(3.0 × 10^−7^, 2.3 × 10^−6^). (d) *β*_2_ − *β*_1_: 9.1 × 10^−11^(4.8 × 10^−11^, 1.5 × 10^−10^), *β*_3_ − *β*_1_: 7.5 × 10^−11^(1.8 × 10^−11^, 1.6 × 10^−10^).

To determine whether difference between serotypes may explain some of the observed variation in viral load dynamics seen in Fig 1, we next fit the three serotype-specific models described above to the viral load data. We first consider model SS_*β*_, which allows viral infectivity rates to differ by serotype. Since DENV-2 is thought to have a high replication rate [18], and DENV-2 often causes more severe disease than DENV-1 and DENV-3 [10], we hypothesize that *β*_2_ > (*β*_1_, *β*_3_). Fig 5a shows the serotype-specific density estimates of *β* for this model. These density estimates indicate that (*β*_2_, *β*_3_) > *β*_1_, partially consistent with our hypothesis. Our finding that the replication rate of DENV-3 is as high as DENV-2’s may be accounted for by a study finding that some DENV-3 clades cause high incidence of severe dengue disease [57].

To address whether there is support for serotypes differing in their ability to block interferon signaling, we fit model SS_*q*_ to the viral load data. Based on [21], we expect the strength of the innate immune response *q* to vary by serotype, with *q*_3_ > *q*_1_ > *q*_2_. Fig 5b shows the serotype-specific density estimates of *q* for this model. The results of this model are contrary to expectation, with *q*_3_ estimates significantly lower than the estimates for *q*_1_ or *q*_2_.

Finally, to address whether there is support for serotypes differing in their elicitation of the T-cell immune response, we fit model ${\text{SS}}_{q_{T}}$ to the viral load data. We specifically hypothesize that T-cell response is higher for secondary DENV-2 and DENV-3 infections than secondary DENV-1 infections (q_*T*2_, q_*T*3_) > q_*T*1_. This hypothesis is based on work by Bashyam and coauthors [58] that found that secondary DENV-2 and DENV-3 infections were associated with higher magnitudes of CD8+ T-cell secreted cytokines than were secondary DENV-1 and DENV-4 infections. This suggests that the T- cell response may be higher for secondary DENV-2 and DENV-3 infections than in secondary DENV-1 and DENV-4 infections. Fig 5c shows serotype-specific density estimates of *q*_*T*_ for this model. The model fits are consistent with our hypothesis that q_*T*2_ and q_*T*3_ values exceed the value of q_*T*1_.

Table 2 shows the median log-likelihood and the BIC and DIC values for each of these three models. All three models have higher log-likelihoods than model 1. Based on BIC and DIC, the viral load data best support the model in which serotypes differ in their viral infectivity rates (model SS_*β*_), followed by model ${\text{SS}}_{q_{T}}$. Only these two models are preferred over model 1. Despite these two models’ similarity in likelihood values, the models differ dramatically in their T-cell dynamics (S6 Fig). As expected, T-cell dynamics are similar across serotypes for model SS_*β*_. In contrast, the magnitude of the T-cell response for DENV-2 and DENV-3 is much higher than for DENV-1 under the ${\text{SS}}_{q_{T}}$ model.

Because we did not see peak viral load in many individuals, it was difficult to distinguish between model SS_*β*_ and model ${\text{SS}}_{q_{T}}$, since both models result in serotype-specific differences in viral clearance. To attempt to statistically distinguish between these two models, we again fit models to the subset of the data that contained viral peaks. Table 4 shows the medium log-likelihood, BIC and DIC values for both models. These results show that model *SS*_*β*_ fits the data significantly better than model ${\text{SS}}_{q_{T}}$ based on both BIC and DIC. Serotype-specific density estimates for *β*, when fit to this subset of data, are shown in Fig 5d. Of note, the estimated IP values under model SS_*β*_ do not appear to differ by serotype (results not shown), regardless of whether the model is fit to the entire viral load dataset or only the data containing viral peaks. That incubation periods do not differ by serotype is consistent with findings from [42].

**Fig 6:**
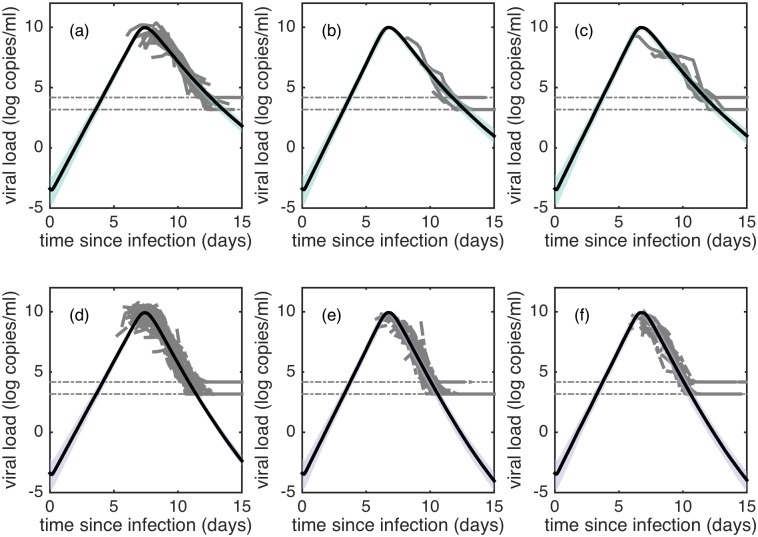
Fits of model SS_*β*_ to viral load data. (a-f) Model simulations alongside viral load measurements from all individuals experiencing a DENV-1 (a,d), DENV-2 (b,e), or DENV-3 (c,f) infection. Primary infection simulations and data are shown in (a-c). Secondary infection simulations and data are shown in (d-f). Gray lines show primary infection data (a-c) and secondary infection data (d-f). Green and purple shaded regions show 95% posterior credible intervals of primary and secondary infections, respectively. Credible intervals were constructed from 100 simulations of model *SS*_*β*_, where parameters are sampled from the posterior for each simulation. Black lines show simulations using median likelihood parameters estimates. In all subplots, dotted horizontal lines show limits of detection. As in Fig 2a and 2b, viremia measurements are shifted in time based on sampled estimates of individual *IP* values.

Fig 6a–6f show simulations of model SS_*β*_ (fit to the entire viral load dataset) alongside viral load data from all individuals experiencing a DENV-1, DENV-2 or DENV-3 infection, stratified by primary infection (Fig 6a–6c) and secondary infection (Fig 6d–6f). The higher viral infectivity rates in DENV-2 and DENV-3 infections relative to DENV-1 infections result in shorter times to peak viremia and higher viral clearance rates. Parameter values for model *SS*_*β*_, when fit to the entire viral load dataset, are given in Table 3. Parameter values for model *SS*_*q*_ and model ${\text{SS}}_{q_{T}}$ fits to the full dataset are provided in S2 Table. Parameter values for model *SS*_*β*_ and model ${\text{SS}}_{q_{T}}$ fits to this subset of the data are provided in S3 Table.

### Models considering differences by clinical manifestation and between dengue serotypes

In our dataset 76% of DENV-1 individuals and 74% of DENV-3 individuals had DF, whereas only 47% of DENV-2 individuals had DF. To ensure that our serotype-specific results were not capturing differences in clinical manifestation, rather than serotype-specific differences, we fit a model (${\text{SS}}_{\beta_{ADE}}$) in which viral infectivity rates were allowed to vary by clinical manifestation and by serotype. We considered this parameter because the ADE model was the best clinical manifestation model to fit the data and model SS_*β*_ was the best serotype-specific model to fit the data.

We expected that, for a given serotype, individuals with DHF would have a higher viral infectivity rate than those with DF, such that: *β*_1,*DHF*_ > *β*_1,*DF*_, *β*_2,*DHF*_ > *β*_2,*DF*_ and *β*_3,*DHF*_ > *β*_3,*DF*_. We also expected, as described above, that individuals with DENV-2 would have the highest viral infectivity rates. Therefore, we would expect that: *β*_2,*DHF*_ > *β*_1,*DHF*_, *β*_3,*DHF*_ and *β*_2,*DF*_ > *β*_1,*DF*_, *β*_3,*DF*_. S7a Fig shows the density estimates of *β* by clinical manifestation and by serotype. The results are similar to those of the *SS*_*β*_ model, with density estimates of *β*_2_, *β*_3_ > *β*_1_ for both DF and DHF viral infectivity rate estimates. Interestingly, there is very little difference in DF and DHF estimates within a given serotype (S7a Fig). Based on BIC and DIC, there is no statistical support to favor this more complex model over model SS_*β*_ (Table 2).

Our previous analysis showed that it is difficult to disentangle a short incubation period from a high viral infectivity rate in the full dataset. Therefore, we fit model ${\text{SS}}_{\beta_{ADE}}$ to the subset of the data in which viral peaks were detected. *β* density estimates are shown in S7b Fig. Based on BIC and DIC, this more complicated model is not preferred over model *SS*_*β*_ (Table 4). Of note, though not statistically significant, density estimates for model ${\text{SS}}_{\beta_{ADE}}$ (S7b Fig) show that the difference between DHF and DF *β* estimates for DENV-2 and DENV-3 are larger than that of DENV-1, providing some evidence for how differences in viral infectivity rates by clinical manifestation vary by serotype.

## Discussion

By fitting within-host dengue models to viral load data, we have shown these data can be used to discriminate between hypothesized within-host processes leading to differences in clinical manifestation and between dengue serotypes.

Our results first indicate that much of the inter-individual variability in dengue viral load dynamics can be explained by differences in the length of individuals’ incubation periods (*IP*). This *IP* variation may arise because of individual differences in viral inoculum sizes, or because of individual differences in the reporting of symptoms. It may also arise because of variation in viral infectivity rates. Indeed, our results indicate that secondary DHF patients generally have shorter *IP*s than do primary and secondary DF patients (Fig 2), consistent with the hypothesis that antibody-dependent enhancement drives disease severity. In contrast, our results, do not support the hypothesis that original antigenic sin of T-cells was a driver of severe disease. Our findings are consistent with recent work indicating that T-cells may have net protective rather than pathologic effects in secondary infections [37, 59].

In previous work by Clapham and coauthors [31], a different dengue within-host model was fit to the DENV-1 viral load data analyzed here. The authors also found differences in the viral infectivity rate *β* by clinical manifestation (primary infection DF, secondary infection DF and secondary infection DHF), with *β*_2^*o*^, *DHF*_ > *β*_2^*o*^, *DF*_ > *β*_1^*o*^, *DF*_, providing evidence for ADE. In their most recent work in which they fit more complicated models to DENV-1 and DENV-2 viral load and antibody data, they found that the model that best fit the data was one in which they assumed antibodies directly neutralize free virus, as opposed to a model in which antibodies indirectly kill infected cells via ADCC. However, they found that the estimated infected cell lifespan of this model was low, suggesting that another viral clearance mechanism, such as T-cells, as we found here, may also be needed to clear the infection.

As in [31], we made use of traditional dengue disease classifications, namely that of DF and DHF/DSS, when fitting our clinical manifestation models to viral load data. DHF/DSS can also be further subclassified into four grades of severity, where grades III and IV correspond to DSS [60]. We expect that if patients manifesting DHF in our dataset were categorized into grades, DHF grade III and IV would have higher viral infectivity rates than DHF grades I and II. This hypothesis would be consistent with a study which showed that maximum viral titer correlated with DHF grade [24], as has been shown in viral kinetic studies where infection is stratified by DF and DHF [23, 25]. The 2009 reclassification of dengue disease into the broader categories of dengue without warning signs, dengue with warning signs and severe dengue can result in more infections classified as severe than the older classifications, and can obstruct serotype-specific associations with specific disease manifestations [60]. For example, in a meta-study of 544 DENV-1-4 infected patients from Nicaragua, with the old classification (DF/DHF/DSS), DENV-2 was significantly associated with DHF/DSS. However, DENV-2 was not significantly associated with severe disease with the new classification [60]. Therefore, our clinical manifestation and serotype-specific results would need to be reevaluated in the context of these broader DENV categories.

In fitting our serotype-specific models to the viral load data, we showed that DENV-1, -2, and -3 differ from one another in their viral infectivity rates *β*, with DENV-2 and DENV-3 having higher viral infectivity rates than DENV-1. This finding stands in contrast to Clapham and coauthors’ work, which did not find differences in DENV-1 and DENV-2 parameter values that contribute to viral clearance, [32], though these differences may be incorporated via inter-individual variation in other parameters. Our own findings are consistent with the hypothesis that serotypes that cause more severe disease may have higher replication rates [18]. Studies have indicated that viral replication rates can vary by dengue genotype, with different genotypes of the same serotype having different replication rates [20, 43]. Unfortunately, the viral load data we have analyzed do not have genotype-level resolution, although during the time period of the clinical trial (2007–2008), DENV-1 genotype I and the Asian-American DENV-2 genotype were dominating [61]. We do not have information about the DENV-3 genotype circulating during this time.

Our serotype-specific findings are consistent with a recent meta-analysis of dengue virus kinetics in non-human primates [62]. This meta-analysis found that the time to detectable viremia for DENV-1 was longer than for DENV-2 and DENV-3, indicating that DENV-1 is likely to have a lower replication rate than either DENV-2 or DENV-3. This finding is also consistent with our results that show DENV-2 and DENV-3 viral load dynamics peak before the onset of symptoms, whereas DENV-1 viral load dynamics peak after the onset of symptoms. These findings provide an explanation for why DENV-1 viral load appears to be high relative to DENV-2 and DENV-3 viral load, despite DENV-1 infections typically resulting in less severe disease [23]. Further, they highlight that measuring viral load magnitude for DENV-2 and DENV-3 infections after the onset of symptoms may not be a good predictor of disease risk because viral clearance has already begun when measurements are typically taken.

Our results are further relevant to understanding serotype-specific differences in population-level transmissibility. For many infectious diseases, viral load levels are thought to affect transmission potential [63]. This is also the case for dengue: the probability of transmitting dengue to *Aedes Aegypti* has been shown to increase with viral load of the infected individual [64]. This suggests that further characterization of serotype-specific differences in viral load dynamics can improve our understanding of dengue virus transmission at the population level.

Our ability to distinguish between models was in part limited by the absence of viral load data early in infection. For example, though shorter time to viral peak and high viremia magnitude is consistently associated with more severe dengue disease [23–25, 56], our models were unable to reproduce these trends because we do not have enough individual viral load data that contained viral peaks. Fitting model ADE to only the viral peak data showed that with data on early infection dynamics we could ultimately discriminate between differences in viral replication rates between individuals differing in clinical manifestation. This model was able to reproduce the earlier time to peak seen during DHF infections relative to DF infections. These results highlight the importance of collecting data before the onset of symptoms in order to detect viral peaks. Additionally, our results highlight that models that result in similar viral dynamics can display very different immune cell dynamics (S6 Fig). Kinetic immune cell data, along with viral load data taken before the onset of symptoms, will be very useful in future work that aims to discriminate between dengue models.

Dengue human infection model experiments currently underway [65] will become an important resource for understanding early viral load and immune cell dynamics. There are currently DENV-2 and DENV-3 viruses that already meet dengue fever criteria for these experiments, and models of all four serotypes are being developed [65]. Though these models have been developed to evaluate vaccine efficacy, infecting naive individuals with these strains and obtaining many viral and immune measurements during the infection will be very useful in deciphering within-host processes leading to severe disease.

A final shortcoming of the viral load data we analyzed is that we do not know the primary infecting serotype for individuals experiencing a secondary heterologous infection. Because order of infection is known to be an important risk factor for severe disease [10], knowledge of the primary infection serotype in further analyses would allow us to disentangle how the primary infection shapes the viral kinetics of secondary heterologous infection. Longitudinal dengue studies in which one dengue serotype typically predominates, such as the Pediatric Dengue Cohort Study in Nicaragua [66], are thus invaluable for ultimately understanding the role that order of infection plays in shaping viral dynamics.

Despite limitations inherent in the data, our analyses indicate that inter-individual variation in dengue viral load patterns can shed light on which within-host processes are important in regulating viral dynamics. Collection of individual dengue viral load data at fine temporal resolution, spanning the entire infection duration, will be important for further understanding the relationships between dengue viral load, disease severity, and dengue transmissibility.

## Supporting Information

S1 Text Full likelihood expression and supplemental details on model 1 results. (PDF)

S1 Fig Traces of model 1. Each color shows a different MCMC run. Every 1000 iterations are shown.
(TIFF)

S2 Fig Correlation structure of model 1. Each subplot shows correlations between two parameter estimates of model 1. Samples are shown for every 100 iterations after burn-in (150,000 iterations).
(TIFF)

S3 Fig Viral load and T-cell dynamics under different assigned *δ*_*T*_ values. (a) Simulated viral load dynamics under different assigned values of *δ*_*T*_. Dotted lines show limits of detection of the assays used. (b) Simulated T-cell dynamics under different assigned values of *δ*_*T*_. In (a) and (b), simulations shown use parameter estimates of *β*, *κ*, *q*, *q*_*T*_, and *V*_0_ that yielded the median posterior. Parameter values used are as follows: *δ*_*T*_ = 10^−5^: *β* = 6.6 × 10^−10^, *κ* = 4.9, *q* = 5.9 × 10^−4^, *q*_*T*_ = 5.9 × 10^−7^, *log*(*V*_0_) = −6.1. *δ*_*T*_ = 10^−6^: *β* = 4.6 × 10^−10^, *κ* = 4.8, *q* = 6.6 × 10^−4^, *q*_*T*_ = 1 × 10^−6^, *log*(*V*_0_) = −3.3. *δ*_*T*_ = 10^−7^: *β* = 4.1 × 10^−10^, *κ* = 5.1, *q* = 6.1 × 10^−4^, *q*_*T*_ = 1.3 × 10^−6^, *log*(*V*_0_) = −2.2. *δ*_*T*_ = 10^−8^: *β* = 3.9 × 10^−10^, *κ* = 5.3, *q* = 8.2 × 10^−4^, *q*_*T*_ = 2.1 × 10^−6^, *log*(*V*_0_) = −1.9.
(TIFF)

S4 Fig Simulations of model 1 fit to individuals who received placebo or chloroquine. Black solid lines and dashed lines show simulations of model 1 for placebo and chloroquine-treated groups, respectively, using median likelihood estimates. Red and yellow shaded regions show 95% posterior credible intervals for placebo and chloroquine-treated groups, respectively (orange regions show overlap of credible regions). Credible intervals were constructed from 100 simulations of each model, where parameters are sampled from the posterior for each simulation. (a) Dynamics of free virus *V* during primary infections. (b) Dynamics of free virus *V* during secondary infections.
(TIFF)

S5 Fig Simulations of model ADE fit to viral peak data, using draws from the posterior distribution. Black solid, short-dashed lines and long-dashed lines show simulations of primary DF, secondary DF and secondary DHF infections, respectively, using median likelihood estimates. Green, orange, and blue shaded regions show 95% posterior credible intervals of primary DF, secondary DF and secondary DHF infections, respectively. Credible intervals were constructed from 100 simulations of model ADE, where parameters are sampled from the posterior for each simulation. (a) Dynamics of uninfected target cells *X*. (b) Dynamics of free virus *V*. (c) Dynamics of NK cells *N*. (d) Dynamics of T-cells *T* in secondary infections.
(TIFF)

S6 FigSimulations of model SS_*β*_ and model ${\text{SS}}_{q_{T}}$’s T-cell dynamics by serotype.(a-c) Model SS_*β*_. (d-f) Model ${\text{SS}}_{q_{T}}$. (a,d) DENV-1 (b,e) DENV-2 (c,f) DENV-3. (a-f) Black solid lines show simulations of secondary infections using median likelihood estimates. Shaded regions show 95% posterior credible intervals of secondary infections by serotype. Credible intervals were constructed from 100 simulations of each model, where parameters are sampled from the posterior for each simulation.(TIFF)

S7 FigParameter estimates from the models in which *β* varies by clinical manifestation and by serotype.(a) Density estimates for clinical manifestation-specific and serotype-specific viral infectivity rates *β* for model ${\text{SS}}_{\beta_{ADE}}$ fit to full dataset. (b) Density estimates for clinical manifestation-specific and serotype-specific viral infectivity rates *β* for model ${\text{SS}}_{\beta_{ADE}}$ fit to peak viral load data subset. (a-b). Solid lines show DF estimates and dotted lines show DHF estimates. DENV-1 estimates are shown in light blue, DENV-2 estimates are shown in dark blue and DENV-3 estimates are shown in green. The median parameter estimate and 95% posterior credible interval (in parentheses) of the difference of *β*_1_, *β*_2_ and *β*_3_ estimates by clinical manifestation for each model are: (a) $\beta_{1_{DHF}}-\beta_{1_{DF}}$: 6.8 × 10^−12^ (4.5 × 10^−13^, 2.7 × 10^−11^). $\beta_{2_{DHF}}-\beta_{2_{DF}}$: 2.0 × 10^−11^ (4.2 × 10^−12^, 5.6 × 10^−11^). $\beta_{3_{DHF}}-\beta_{3_{DF}}$: 2.6 × 10^−11^(5.3 × 10^−12^, 8.0 × 10^−11^). (b) $\beta_{1_{DHF}}-\beta_{1_{DF}}$: 2.9 × 10^−11^ (3.2 × 10^−12^, 7.4 × 10^−11^). $\beta_{2_{DHF}}-\beta_{2_{DF}}$: 8.5 × 10^−11^ (2.5 × 10^−11^, 2.0 × 10^−10^). $\beta_{3_{DHF}}-\beta_{3_{DF}}$: 7.8 × 10^−11^ (2.0 × 10^−11^, 2.2 × 10^−10^).(TIFF)

S1 Table Parameter estimates for model 1 fit to individuals who received placebo or chloroquine. Median marginal posterior parameter estimates are reported, with 95% posterior credible intervals for each parameter in parentheses. Units are the same as in Table 1 in the main text.
(PDF)

S2 Table Parameter estimates for models fit to full dataset. Median marginal posterior parameter estimates are reported, with 95% posterior credible intervals for each parameter in parentheses. Units are the same as in Table 1 in the main text.
(PDF)

S3 Table Parameter estimates for models fit to subset of data with detected viral peaks. Median marginal posterior parameter estimates are reported, with 95% posterior credible intervals for each parameter in parentheses. Units are the same as in Table 4 in the main text. *V*_0_ = 10^−3.2^ copies/cell.
(PDF)

S4 Table Model comparisons when *X*_0_ is varied 1/2 and 2 times its set point estimate used in Table 1 in the main text. Median log-likelihood values, BIC and DIC values for all models considered are reported.
(PDF)

S5 Table Model comparisons when *T*_0_ is varied 1/2 and 2 times its set point estimate used in Table 1 in the main text. Median log-likelihood values, BIC and DIC values for all models considered are reported.
(PDF)

S6 Table Model comparisons when *ω* is varied 1/2 and 2 times its set point estimate used in Table 1 in the main text. Median log-likelihood values, BIC and DIC values for all models considered are reported.
(PDF)

S7 Table Model comparisons when 1/*d* is varied 1/2 and 2 times its set point estimate used in Table 1 in the main text. Median log-likelihood values, BIC and DIC values for all models considered are reported.
(PDF)

S8 Table Model comparisons when 1/*d*_*T*_ is varied 1/2 and 2 times its set point estimate used in Table 1 in the main text. Median log-likelihood values, BIC and DIC values for all models considered are reported.
(PDF)
