# Supplementary material for: Drivers of Inter-individual Variation in Dengue Viral Load Dynamics
Source: PLoS Comput Biol. 2016 Nov 17;12(11):e1005194. doi: 10.1371/journal.pcbi.1005194 (PMC5113863; doi:10.1371/journal.pcbi.1005194)
Supplement: S1 Text — (PDF) [file pcbi.1005194.s001.pdf]

S1 Text: Full likelihood expression and supplemental details on model 1 results

Full likelihood expression

The full likelihood expression is given by:

$$L(\gamma|D) = \prod_{j=1}^{N} \int_{IP_j=a}^{b} (\prod_{k=1}^{n} \phi(D_j(t_k)|M(\gamma, t_k + IP_j), \sigma_\epsilon) \mathbb{1}_{D_{j(t_k)} > LOD} + \Phi(LOD|M(\gamma, t_k + IP_j), \sigma_\epsilon) \mathbb{1}_{D_{j(t_k)} \leq LOD}) dIP_j \quad (1)$$

where a and b are the 0.1 and 99.9 percentiles of $logN(log(IP_g), \sigma_I)$ and N is the total number of individuals.

Supplemental details on model 1 results

When fitting equations (1) to viral load measurements from individuals experiencing secondary infections, we found that the rate at which T-cells clear infected cells (δ_T) could not be accurately estimated. To determine why this might be the case, we set δ_T to a given value and estimated the remaining model parameters (β, κ, q, and q_T) and initial condition (V_0). Fig. S3a shows viral load dynamics simulated using median likelihood parameter estimates for 4 different values we assigned to $\delta_T(10^{-5}$/day, 10^{-6}/day, 10^{-7}/day, and 10^{-8}/day). Fig. S3b shows the simulated T-cell dynamics under these assigned values of δ_T, indicating that the value of δ_T considerably impacted T-cell dynamics. Simulated viral load dynamics (Fig. S3a), however, were remarkably similar to one another during viral decline, the period for which we have data. Because immunological studies examining T-cell dynamics have found that T-cells reach counts on the order of 10^6 cells/ml in dengue infections [1, 2], we set δ_T to 10^{-6} per day (Fig. S3b).

Using model 1, we further re-evaluated whether chloroquine treatment had a measurable effect on viral load dynamics. Of the 228 patients analyzed in our analysis, 112 patients received a placebo and 116 received chloroquine treatment. Of the individuals who received a placebo, 9 (8%) were experiencing a primary infection and 103 (92%) were experiencing a secondary infection. 62 (55.4%) of the placebo recipients were infected with DENV-1, 25 (22.3%) with DENV-2 and 25 (22.3%) with DENV-3. Of the individuals who received chloroquine, 17 (15%) were experiencing a primary infection and 99 (85%) were experiencing a secondary infection. 77 (66%) of the chloroquine recipients were infected with DENV-1, 25 (22%) with DENV-2 and 14 (12%) with DENV-3. Parameter estimates resulting from fitting model 1 individually to chloroquine-treated and placebo individuals are shown in S1 Table, with 95% posterior credible intervals of parameter estimates overlapping one another. Fig. S4 further shows simulations of model 1 for placebo and chloroquine-treated groups, for primary as well as secondary infections, visually indicating that these groups did not differ systematically in their viral load dynamics. Both the placebo and the chloroquine-treated group exhibited a steeper viral decline in secondary infections compared to primary infections, reproducing the results for model 1 fit to all individuals at once.

References

1. de Matos AM, Carvalho KI, Rosa DS, Villas-Boas LS, da Silva WC, Rodrigues CLdL, et al. CD8+ T Lymphocyte Expansion, Proliferation and Activation in Dengue Fever. PLoS Negl Trop Dis. 2015 Feb;9(2):e0003520.

2. Green S, Vaughn DW, Kalayanarooj S, Nimmannitya S, Suntayakorn S, Nisalak A, et al. Early immune activation in acute dengue illness is related to development of plasma leakage and disease severity. J Infect Dis. 1999 Apr;179(4):755–62.
