# Supplementary figures and images for: Drivers of Inter-individual Variation in Dengue Viral Load Dynamics
Source: PLoS Comput Biol. 2016 Nov 17;12(11):e1005194. doi: 10.1371/journal.pcbi.1005194 (PMC5113863; doi:10.1371/journal.pcbi.1005194)

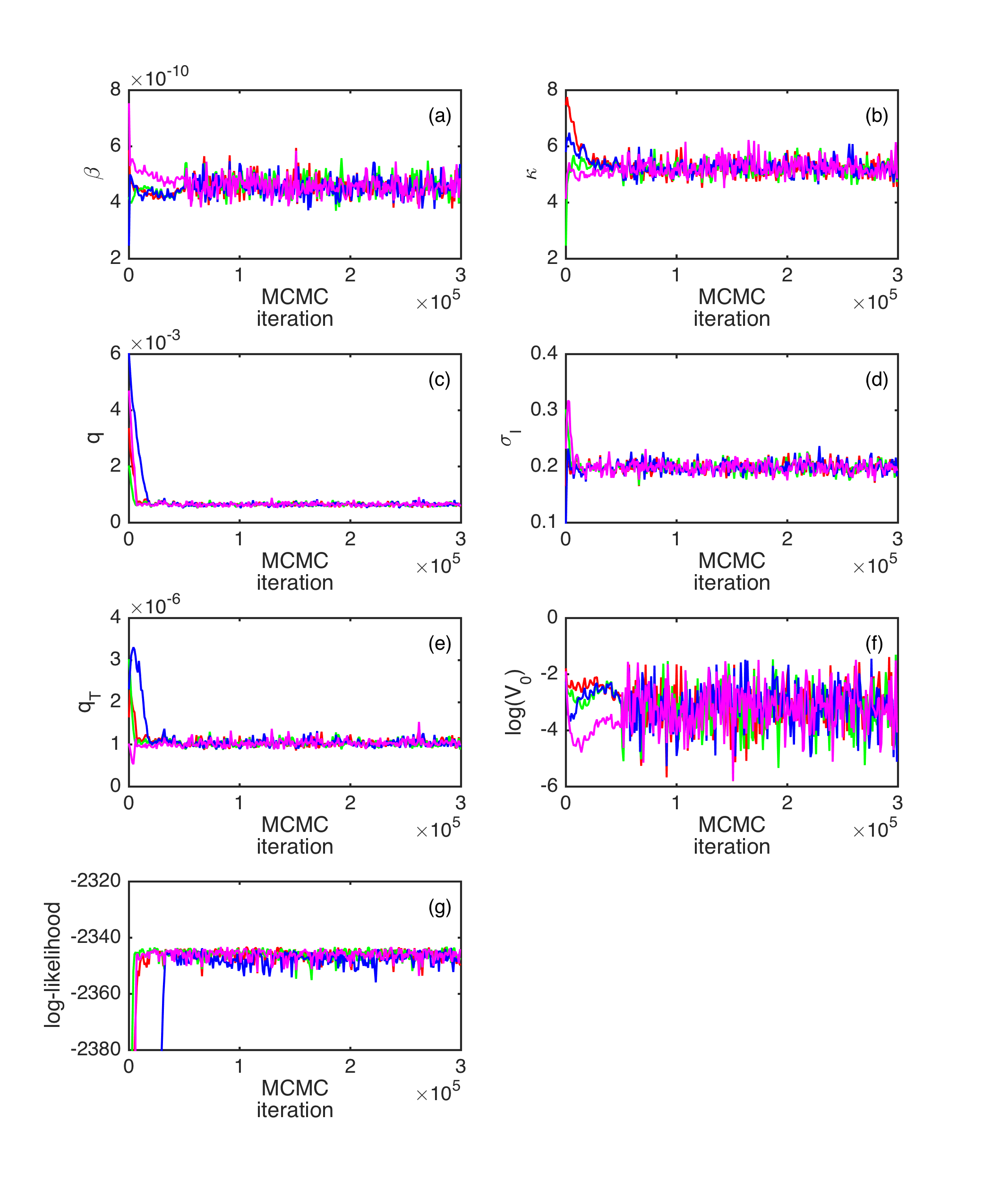

Supplement: S1 Fig — Each color shows a different MCMC run. Every 1000 iterations are shown. (TIFF) [file pcbi.1005194.s002.tiff]

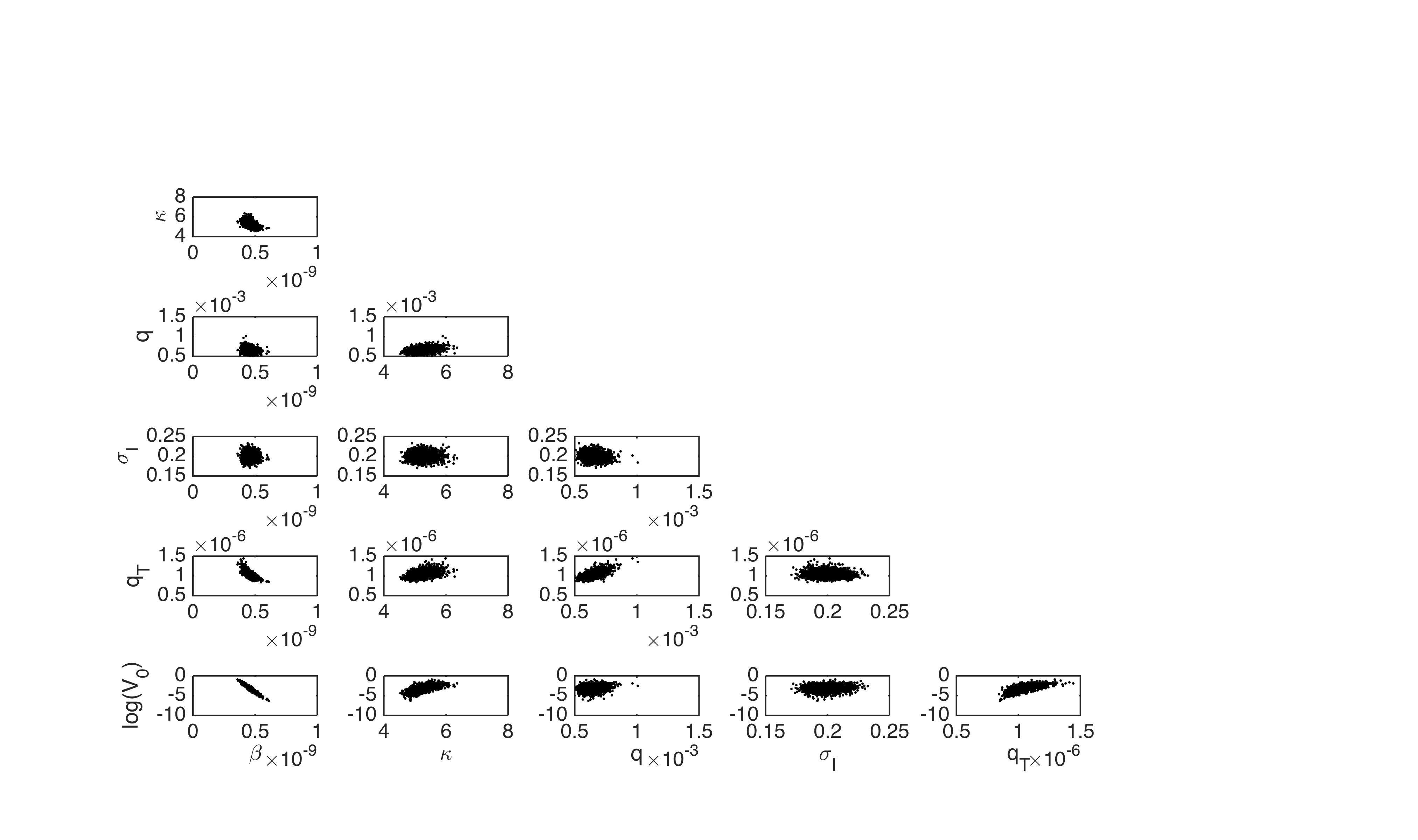

Supplement: S2 Fig — Each subplot shows correlations between two parameter estimates of model 1. Samples are shown for every 100 iterations after burn-in (150,000 iterations). (TIFF) [file pcbi.1005194.s003.tiff]

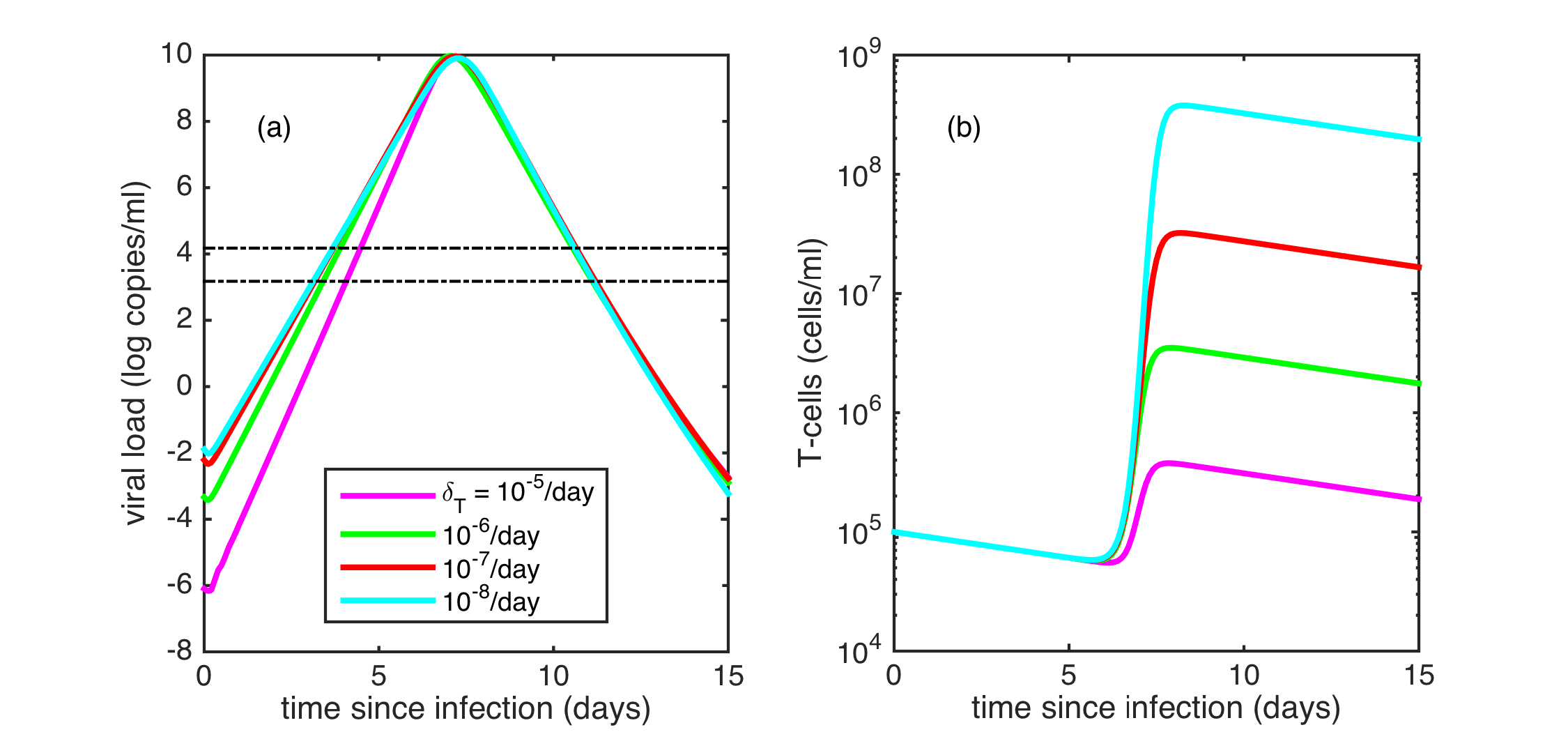

Supplement: S3 Fig — (a) Simulated viral load dynamics under different assigned values of δT. Dotted lines show limits of detection of the assays used. (b) Simulated T-cell dynamics under different assigned values of δT. In (a) and (b), simulations shown use parameter estimates of β, κ, q, qT, and V0 that yielded the median posterior. Parameter values used are as follows: δT = 10−5: β = 6.6 × 10^−10^, κ = 4.9, q = 5.9 × 10^−4^, qT = 5.9 × 10^−7^, log(V0) = −6.1. δT = 10−6: β = 4.6 × 10^−10^, κ = 4.8, q = 6.6 × 10^−4^, qT = 1 × 10^−6^, log(V0) = −3.3. δT = 10−7: β = 4.1 × 10^−10^, κ = 5.1, q = 6.1 × 10^−4^, qT = 1.3 × 10^−6^, log(V0) = −2.2. δT = 10−8: β = 3.9 × 10^−10^, κ = 5.3, q = 8.2 × 10^−4^, qT = 2.1 × 10^−6^, log(V0) = −1.9. (TIFF) [file pcbi.1005194.s004.tiff]

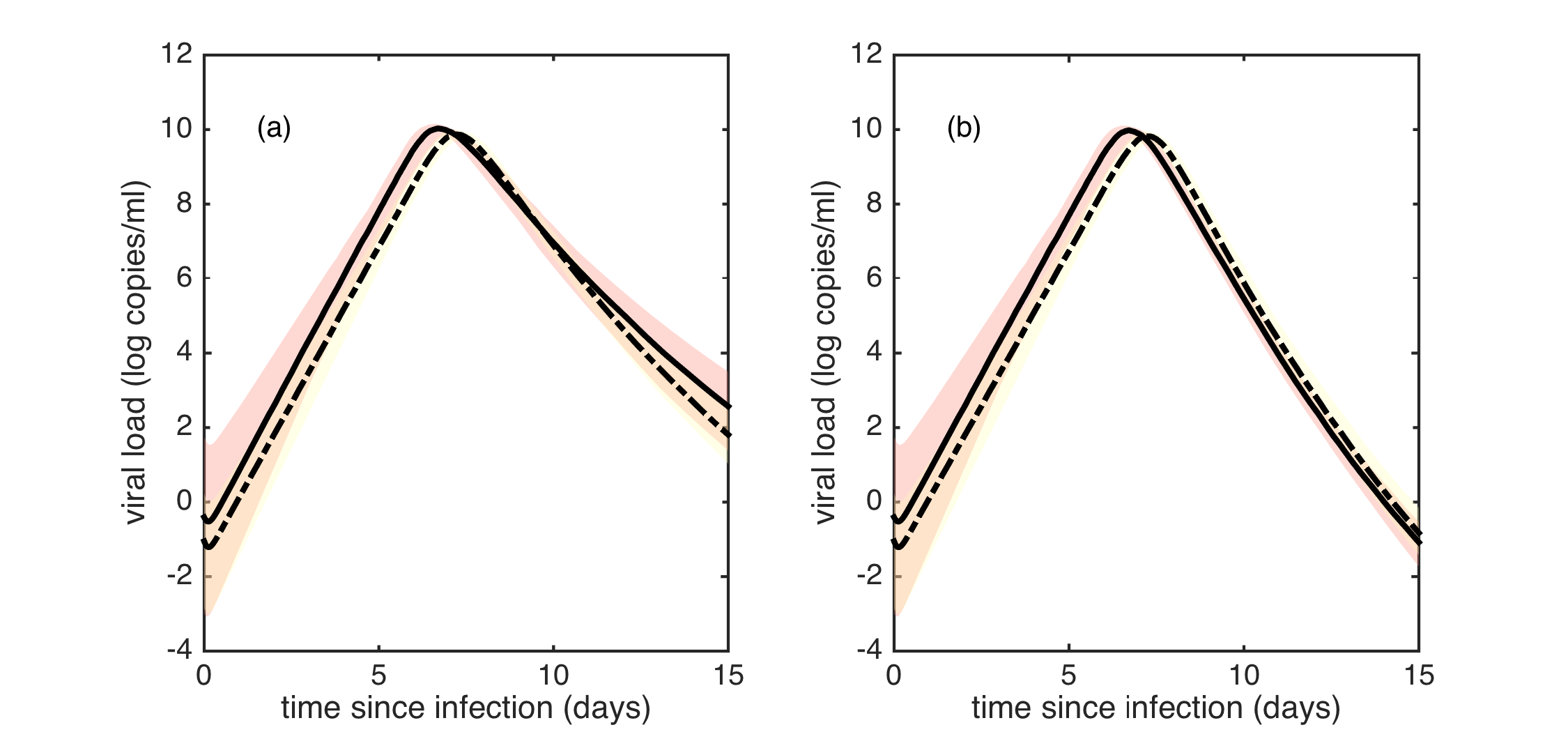

Supplement: S4 Fig — Black solid lines and dashed lines show simulations of model 1 for placebo and chloroquine-treated groups, respectively, using median likelihood estimates. Red and yellow shaded regions show 95% posterior credible intervals for placebo and chloroquine-treated groups, respectively (orange regions show overlap of credible regions). Credible intervals were constructed from 100 simulations of each model, where parameters are sampled from the posterior for each simulation. (a) Dynamics of free virus V during primary infections. (b) Dynamics of free virus V during secondary infections. (TIFF) [file pcbi.1005194.s005.tiff]

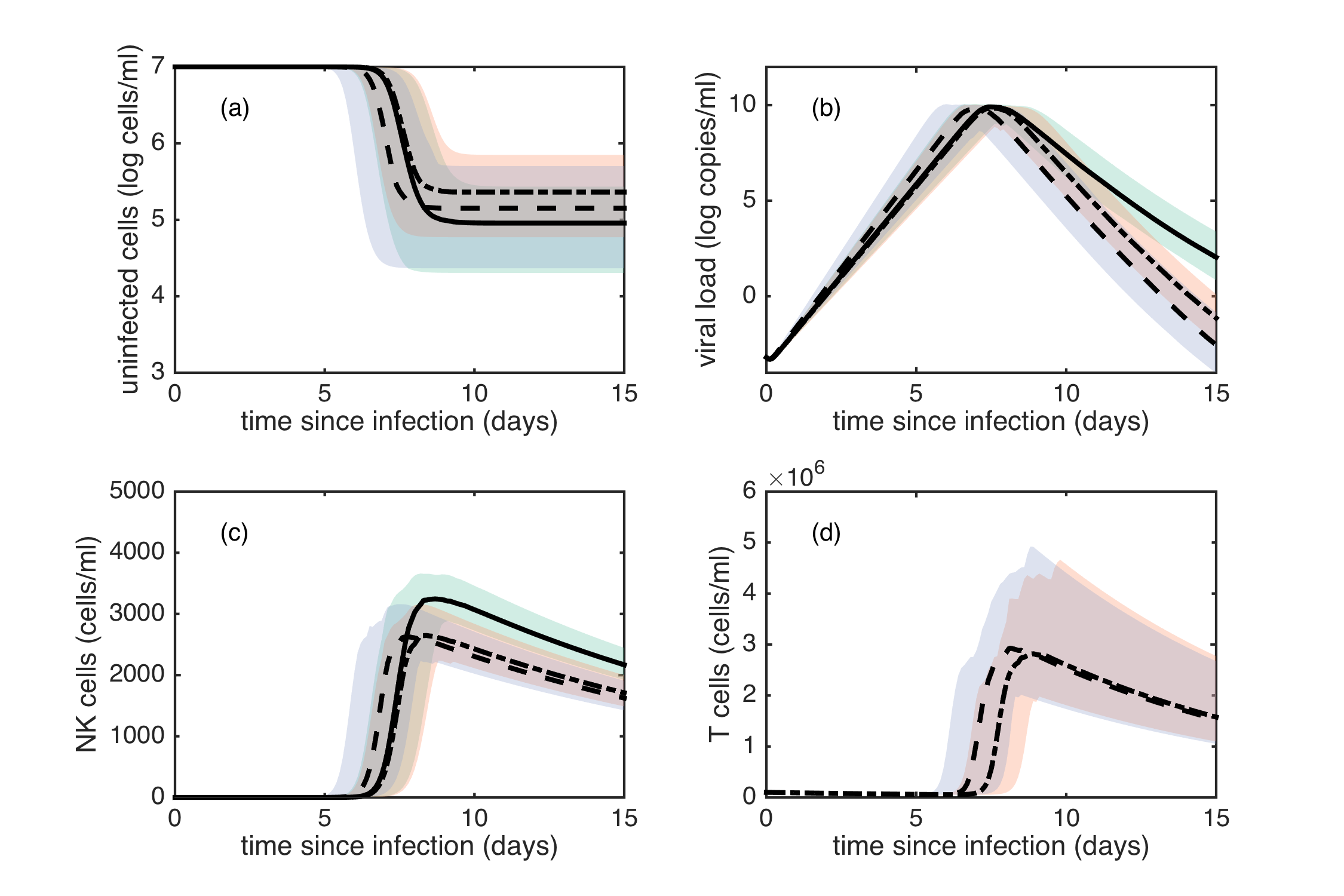

Supplement: S5 Fig — Black solid, short-dashed lines and long-dashed lines show simulations of primary DF, secondary DF and secondary DHF infections, respectively, using median likelihood estimates. Green, orange, and blue shaded regions show 95% posterior credible intervals of primary DF, secondary DF and secondary DHF infections, respectively. Credible intervals were constructed from 100 simulations of model ADE, where parameters are sampled from the posterior for each simulation. (a) Dynamics of uninfected target cells X. (b) Dynamics of free virus V. (c) Dynamics of NK cells N. (d) Dynamics of T-cells T in secondary infections. (TIFF) [file pcbi.1005194.s006.tiff]

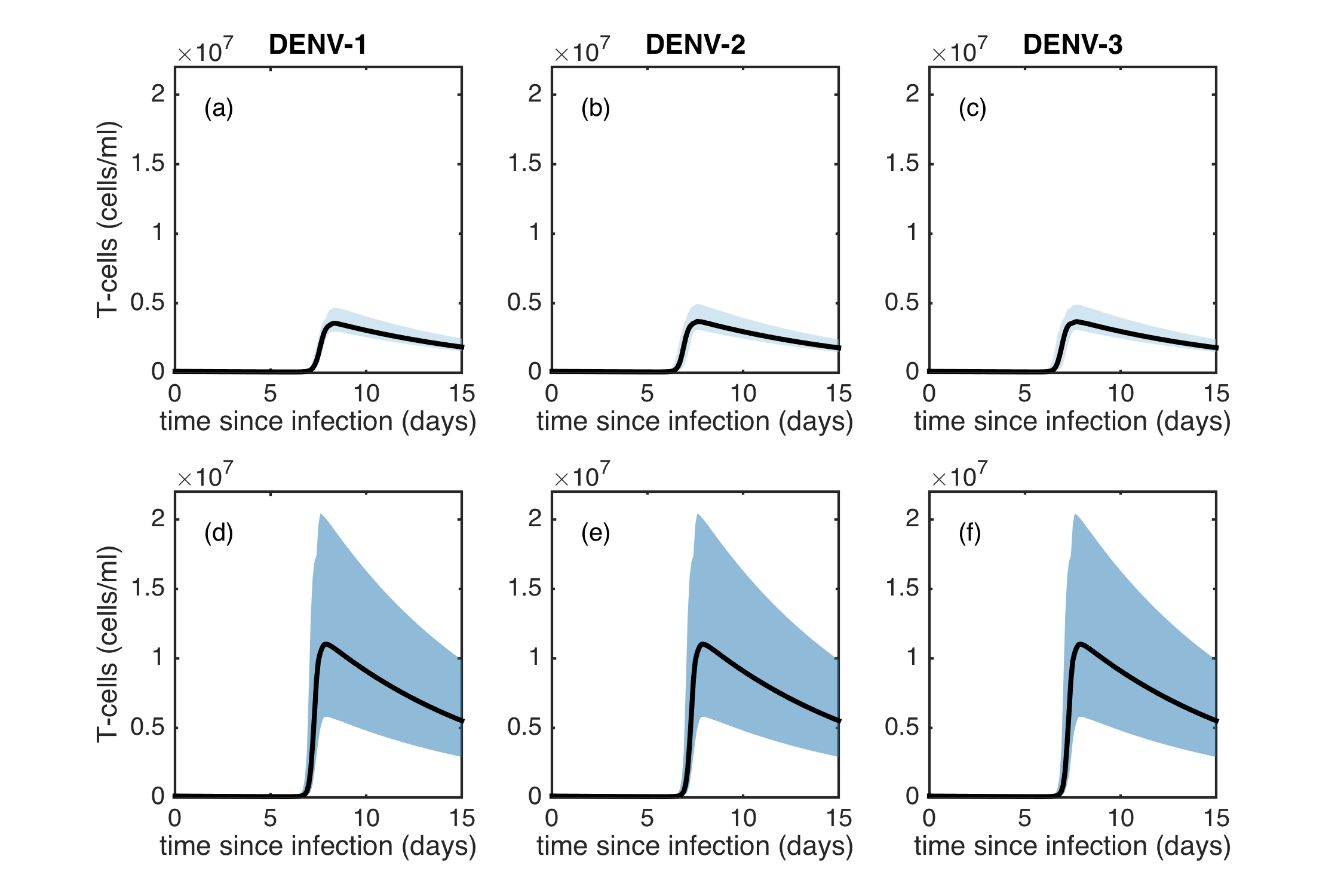

Supplement: S6 Fig — (a-c) Model SSβ. (d-f) Model SSqT. (a,d) DENV-1 (b,e) DENV-2 (c,f) DENV-3. (a-f) Black solid lines show simulations of secondary infections using median likelihood estimates. Shaded regions show 95% posterior credible intervals of secondary infections by serotype. Credible intervals were constructed from 100 simulations of each model, where parameters are sampled from the posterior for each simulation. (TIFF) [file pcbi.1005194.s007.tiff]

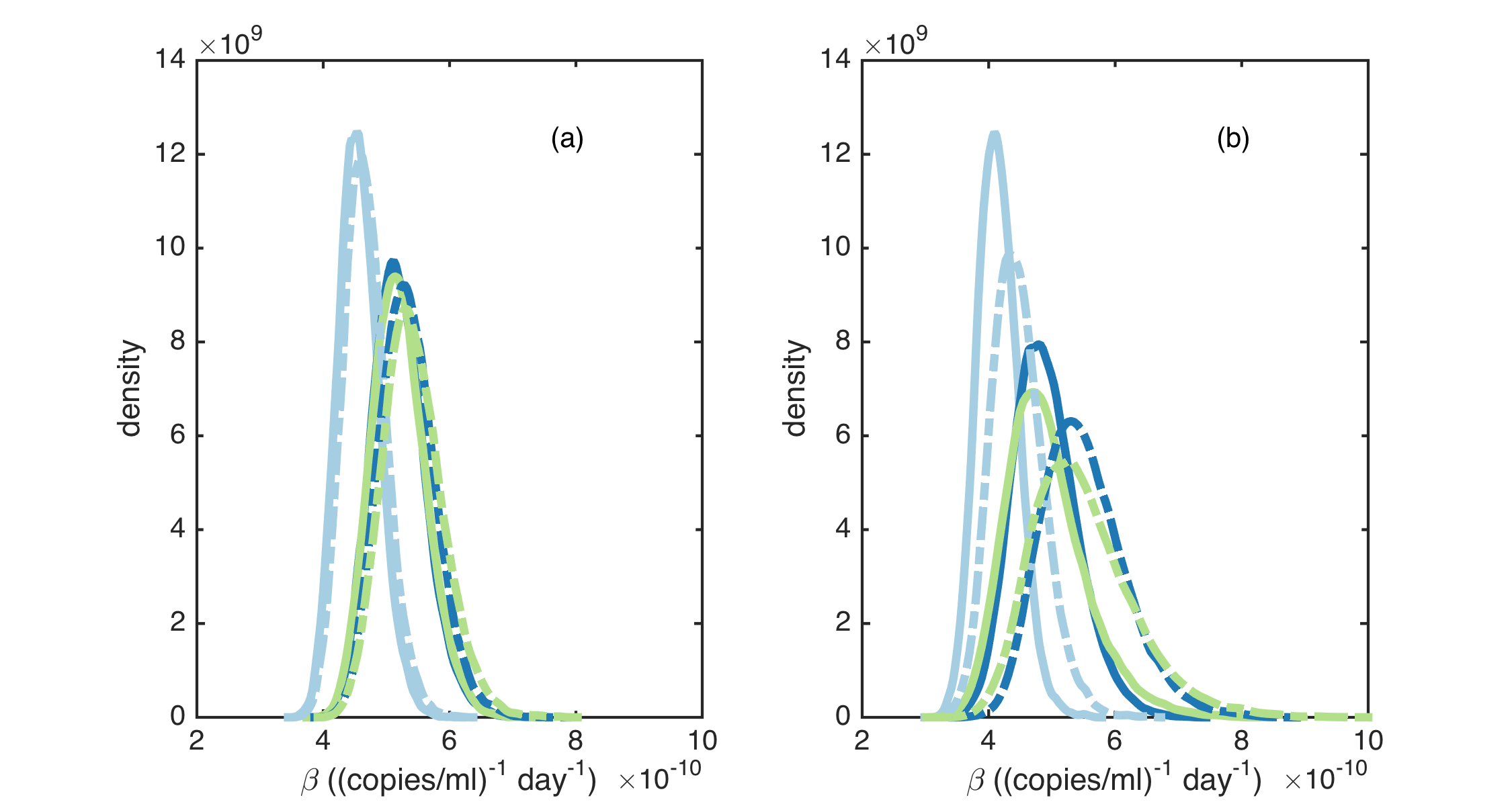

Supplement: S7 Fig — (a) Density estimates for clinical manifestation-specific and serotype-specific viral infectivity rates β for model SSβADE fit to full dataset. (b) Density estimates for clinical manifestation-specific and serotype-specific viral infectivity rates β for model SSβADE fit to peak viral load data subset. (a-b). Solid lines show DF estimates and dotted lines show DHF estimates. DENV-1 estimates are shown in light blue, DENV-2 estimates are shown in dark blue and DENV-3 estimates are shown in green. The median parameter estimate and 95% posterior credible interval (in parentheses) of the difference of β1, β2 and β3 estimates by clinical manifestation for each model are: (a) β1DHF−β1DF: 6.8 × 10^−12^ (4.5 × 10^−13^, 2.7 × 10^−11^). β2DHF−β2DF: 2.0 × 10^−11^ (4.2 × 10^−12^, 5.6 × 10^−11^). β3DHF−β3DF: 2.6 × 10^−11^(5.3 × 10^−12^, 8.0 × 10^−11^). (b) β1DHF−β1DF: 2.9 × 10^−11^ (3.2 × 10^−12^, 7.4 × 10^−11^). β2DHF−β2DF: 8.5 × 10^−11^ (2.5 × 10^−11^, 2.0 × 10^−10^). β3DHF−β3DF: 7.8 × 10^−11^ (2.0 × 10^−11^, 2.2 × 10^−10^). (TIFF) [file pcbi.1005194.s008.tiff]
