# Supplementary material for: Drivers of Inter-individual Variation in Dengue Viral Load Dynamics
Source: PLoS Comput Biol. 2016 Nov 17;12(11):e1005194. doi: 10.1371/journal.pcbi.1005194 (PMC5113863; doi:10.1371/journal.pcbi.1005194)
Supplement: S1 Table — Median marginal posterior parameter estimates are reported, with 95% posterior credible intervals for each parameter in parentheses. Units are the same as in Table 1 in the main text. (PDF) [file pcbi.1005194.s009.pdf]

S1 Table: Parameter estimates for model 1 fit to individuals who received placebo or chloroquine. Median marginal posterior parameter estimates are reported, with 95 % posterior credible intervals for each parameter in parentheses. Units are the same as in Table 1 in the main text.

**Model**	log V_0	$\beta(\times 10^{-10})$	κ	$q(\times 10^{-4})$	$q_T(\times 10^{-6})$	log σ_I
Placebo	-0.46 (-3.7, 1.6)	3.5 (2.7, 5.0)	4.6 (3.8, 6.1)	4.8 (3.5, 7.3)	.81 (.65, 1.1)	.21 (.18, .24)
Chloroquine	-1.1 (-3.1, 0.34)	3.6 (3.0, 4.7)	5.4 (4.4, 7.1)	7.6 (5.4, 11.0)	1.0 (.74, 1.5)	.18 (.16, .21)
