# Supplementary material for: Drivers of Inter-individual Variation in Dengue Viral Load Dynamics
Source: PLoS Comput Biol. 2016 Nov 17;12(11):e1005194. doi: 10.1371/journal.pcbi.1005194 (PMC5113863; doi:10.1371/journal.pcbi.1005194)
Supplement: S2 Table — Median marginal posterior parameter estimates are reported, with 95% posterior credible intervals for each parameter in parentheses. Units are the same as in Table 1 in the main text. (PDF) [file pcbi.1005194.s010.pdf]

S2 Table: Parameter estimates for models fit to full dataset. Median marginal posterior parameter estimates are reported, with 95 % posterior credible intervals for each parameter in parentheses. Units are the same as in Table 1 in the main text.

**Model**	log V_0	$\beta(\times 10^{-10})$	κ	$q(\times 10^{-4})$	$q_T(\times 10^{-6})$	log σ_I	$\delta_{T_{DF}}(\times 10^{-6})$
0	-7.7 (-8.9, -6.6)	$6.7(6.2, 7.3)$	4.6 (4.3, 4.9)	$14(13, 16)$	–	.20 (.18, .22)	–
OAS_1	-3.1 (-4.7, -1.8)	4.5 (4, 5.2)	5.2 (4.8, 5.8)	6.5 (5.5, 7.75)	1.0 (.90, 1.2)	.20 (.18, .22)	1.1 (1.0, 1.7)
OAS_2	-3.2 (-4.8, -1.8)	4.6 (4.0, 5.3)	5.2 (4.8, 5.9)	6.5 (5.5, 7.7)	DF: .97 (.83, 1.2)<br>DHF: 1.1 (0.95, 1.4)	.20 (.18, .22)	1.2 (1.0, 2.1)
ADE	-3.3 (-4.9, -1.9)	DF: 4.6 (4.0, 5.3)<br>DHF: 4.7 (4.1, 5.5)	5.2 (4.8, 5.9)	6.5 (5.5, 7.8)	1.0 (.90, 1.2)	.2 (.18, .22)	–
SS_q	-3.3 (-5.4, -1.8)	4.6 (4.0, 5.6)	5.2 (4.7, 5.9)	q_1: 6.7 (5.4, 8.5)<br>q_2: 7.3 (5.7, 10)<br>q_3: 5.4 (4.2, 7.5)	1 (8.8, 1.2)	0.2 (.18, .22)	–
SS_{q_T}	-2.9 (-4.3, -1.7)	4.3 (3.9, 4.9)	5.3 (4.9, 5.8)	6.9 (5.8, 8.4)	q_{T_1}: 1.0 (.9, 1.2)<br>q_{T_2}: 1.7 (1.1, 3.2)<br>q_{T_3}: 2.0 (1.3, 3.4)	.19 (.17, .21)	–
$SS_{\beta_{ADE}}$	-3.7 (-5.3, -2.3)	$\beta_{1_{DF}}$: 4.5 (4.0, 5.3)<br>$\beta_{2_{DF}}$: 5.2 (4.4, 6.1)<br>$\beta_{3_{DF}}$: 5.2 (4.4, 6.1)<br>$\beta_{1_{DHF}}$: 4.6 (4.0, 5.4)<br>$\beta_{2_{DHF}}$: 5.3 (4.6, 6.3)<br>$\beta_{3_{DHF}}$: 5.4 (4.6, 6.4)	5.1 (4.7, 5.7)	6.6 (5.6, 7.9)	1.0 (.9, 1.2)	.19 (.17, .21)	–
