# Supplementary material for: Drivers of Inter-individual Variation in Dengue Viral Load Dynamics
Source: PLoS Comput Biol. 2016 Nov 17;12(11):e1005194. doi: 10.1371/journal.pcbi.1005194 (PMC5113863; doi:10.1371/journal.pcbi.1005194)
Supplement: S3 Table — Median marginal posterior parameter estimates are reported, with 95% posterior credible intervals for each parameter in parentheses. Units are the same as in Table 4 in the main text. V0 = 10−3.2 copies/cell. (PDF) [file pcbi.1005194.s011.pdf]

S3 Table: Parameter estimates for models fit to subset of data with detected viral peaks. Median marginal posterior parameter estimates are reported, with 95 % posterior credible intervals for each parameter in parentheses. Units are the same as in Table 4 in the main text. $V_0 = 10^{-3.2}$ copies/cell.

**Model**	IP_g	$\beta(\times 10^{-10})$	κ	$q(\times 10^{-4})$	$q_T(\times 10^{-6})$	$\log \sigma_I$
1	5.8 (5.0, 6.8)	4.0(3.5, 4.7)	4.7 (4.3, 5.4)	6.2(4.9, 8.1)	.97 (.82, 1.2)	.15 (.12, .19)
ADE	5.6 (4.8, 6.5)	DF: 4.0 (3.5, 4.7)<br>DHF: 4.5 (3.8, 5.5)	4.7 (4.3, 5.4)	6.5 (5.2, 8.6)	.95 (.81, 1.2)	.15 (.12, .18)
SS$_\beta$	5.6 (4.9, 6.5)	β_1: 3.9 (3.4, 4.6)<br>β_2: 4.9 (4.0, 6.0)<br>β_3: 4.7 (3.8, 5.9)	4.8 (4.3, 5.4)	6.5 (5.1, 8.5)	1.0 (.08, 1.2)	.14 (.11, .18)
SS$_{q_T}$	5.8 (5.0, 6.7)	4.0 (3.4, 4.6)	4.9 (4.5, 5.5)	6.7 (5.3, 8.8)	q_{T_1}: .94 (.80, 1.2)<br>q_{T_2}: 2.5 (1.3, 6.2)<br>q_{T_3}: 4.1 (1.3, 12)	.14 (.12, .18)
SS$_{\beta_{ADE}}$	5.3 (4.5, 6.1)	$\beta_{1_{DF}}$: 4.1 (3.6, 4.8)<br>$\beta_{2_{DF}}$: 4.9 (4.1, 5.9)<br>$\beta_{3_{DF}}$: 4.8 (3.9, 6.4)<br>$\beta_{1_{DHF}}$: 4.4 (3.7, 5.4)<br>$\beta_{2_{DHF}}$: 5.7 (4.6, 7.4)<br>$\beta_{3_{DHF}}$: 5.7 (4.4, 7.8)	4.6 (4.2, 5.3)	6.7 (5.3, 8.7)	.09 (.08, 1.1)	.15 (.12, .19)
