# Supplementary material for: Drivers of Inter-individual Variation in Dengue Viral Load Dynamics
Source: PLoS Comput Biol. 2016 Nov 17;12(11):e1005194. doi: 10.1371/journal.pcbi.1005194 (PMC5113863; doi:10.1371/journal.pcbi.1005194)
Supplement: S4 Table — Median log-likelihood values, BIC and DIC values for all models considered are reported. (PDF) [file pcbi.1005194.s012.pdf]

Table S4: Model comparisons when X_0 is varied $1/2$ and 2 times its set point estimate used in Table 1 in the main text. Median log-likelihood values, BIC and DIC values for all models considered are reported.

**Model**	**Log-likelihood**	**BIC**	**DIC**
Low value: $X_0 = 5 \times 10^6$ cells/ml			
0	-2452	4938	4909
1	-2376	4793	4758
OAS_1	-2377	4801	4760
OAS_2	-2376	4806	4760
ADE	-2376	4800	4759
SS_β	-2362	4778	4731
SS_q	-2376	4806	4760
SS_{q_T}	-2366	4787	4740
$\mathrm{SS}_{\beta_{ADE}}$	-2364	4803	4737
High value: $X_0 = 2 \times 10^7$ cells/ml			
0	-2393	4821	4792
1	-2336	4714	4678
OAS_1	-2337	4722	4680
OAS_2	-2337	4728	4679
ADE	-2336	4721	4679
SS_β	-2324	4703	4655
SS_q	-2334	4724	4677
SS_{q_T}	-2326	4708	4661
$\mathrm{SS}_{\beta_{ADE}}$	-2326	4727	4660
