# Supplementary material for: Drivers of Inter-individual Variation in Dengue Viral Load Dynamics
Source: PLoS Comput Biol. 2016 Nov 17;12(11):e1005194. doi: 10.1371/journal.pcbi.1005194 (PMC5113863; doi:10.1371/journal.pcbi.1005194)
Supplement: S5 Table — Median log-likelihood values, BIC and DIC values for all models considered are reported. (PDF) [file pcbi.1005194.s013.pdf]

S5 Table: Model comparisons when T_0 is varied $1/2$ and 2 times its set point estimate used in Table 1 in the main text. Median log-likelihood values, BIC and DIC values for all models considered are reported.

**Model**	**Log-likelihood**	**BIC**	**DIC**
Low value: $T_0 = 5 \times 10^4$ cells/ml			
1	-2341	4857	4723
OAS_1	-2342	4731	4690
OAS_2	-2341	4736	4690
ADE	-2376	4731	4689
SS_β	-2328	4711	4663
SS_q	-2340	4735	4688
SS_{q_T}	-2333	4720	4672
$\mathrm{SS}_{\beta_{ADE}}$	-2330	4735	4669
High value: $T_0 = 2 \times 10^5$ cells/ml			
1	-2352	4746	4710
OAS_1	-2353	4754	4713
OAS_2	-2353	4759	4712
ADE	-2352	4753	4711
SS_β	-2338	4732	4685
SS_q	-2352	4758	4711
SS_{q_T}	-2342	4740	4693
$\mathrm{SS}_{\beta_{ADE}}$	-2341	4757	4691
