# Supplementary material for: Drivers of Inter-individual Variation in Dengue Viral Load Dynamics
Source: PLoS Comput Biol. 2016 Nov 17;12(11):e1005194. doi: 10.1371/journal.pcbi.1005194 (PMC5113863; doi:10.1371/journal.pcbi.1005194)
Supplement: S7 Table — Median log-likelihood values, BIC and DIC values for all models considered are reported. (PDF) [file pcbi.1005194.s015.pdf]

S7 Table: Model comparisons when $1/d$ is varied $1/2$ and 2 times its set point estimate used in Table 1 in the main text. Median log-likelihood values, BIC and DIC values for all models considered are reported.

**Model**	**Log-likelihood**	**BIC**	**DIC**
Low value: $d = 0.035$/day			
0	-2396	4826	4797
1	-2334	4709	4674
OAS_1	-2335	4717	4676
OAS_2	-2334	4722	4675
ADE	-2334	4717	4675
SS_β	-2321	4698	4650
SS_q	-2333	4721	4675
SS_{q_T}	-2325	4706	4658
$\text{SS}_{\beta_{ADE}}$	-2324	4722	4656
High value: $d = 0.14$/day			
0	-2444	4922	4893
1	-2374	4789	4754
OAS_1	-2375	4798	4757
OAS_2	-2375	4803	4756
ADE	-2374	4797	4755
SS_β	-2359	4773	4726
SS_q	-2374	4802	4756
SS_{q_T}	-2364	4783	4736
$\text{SS}_{\beta_{ADE}}$	-2361	4797	4732
