# Supplementary material for: Drivers of Inter-individual Variation in Dengue Viral Load Dynamics
Source: PLoS Comput Biol. 2016 Nov 17;12(11):e1005194. doi: 10.1371/journal.pcbi.1005194 (PMC5113863; doi:10.1371/journal.pcbi.1005194)
Supplement: S8 Table — Median log-likelihood values, BIC and DIC values for all models considered are reported. (PDF) [file pcbi.1005194.s016.pdf]

S8 Table: Model comparisons when $1/d_T$ is varied $1/2$ and 2 times its set point estimate used in Table 1 in the main text. Median log-likelihood values, BIC and DIC values for all models considered are reported.

**Model**	**Log-likelihood**	**BIC**	**DIC**
Low value: $d_T = .05$/day			
0	-2411	4857	4828
1	-2342	4725	4690
OAS_1	-2343	4734	4692
OAS_2	-2342	4739	4691
ADE	-2342	4733	4691
SS_β	-2329	4713	4666
SS_q	-2341	4738	4691
SS_{q_T}	-2333	4721	4674
$\mathrm{SS}_{\beta_{ADE}}$	-2331	4738	4671
High value: $d_T = 0.2$/day			
0	-2411	4857	4828
1	-2353	4748	4713
OAS_1	-2355	4757	4716
OAS_2	-2354	4762	4715
ADE	-2354	4756	4715
SS_β	-2339	4734	4687
SS_q	-2353	4761	4714
SS_{q_T}	-2344	4743	4695
$\mathrm{SS}_{\beta_{ADE}}$	-2342	4758	4693
